# Supplementary material for: Double-Weighted Bayesian Model Combination for Metabolomics Data Description and Prediction
Source: Metabolites. 2025 Mar 21;15(4):214. doi: 10.3390/metabo15040214 (PMC12029032; doi:10.3390/metabo15040214)
Supplement: Supplementary file 1 [file metabolites-15-00214-s001.zip › metabolites-3488782-supplementary/Supplementary S1.pdf]

Supplementary S1: List of papers where the DW-EML method was successfully applied to original data.

1. Troisi, J.; Colucci, A.; Cavallo, P.; Richards, S.; Symes, S.; Landolfi, A.; Scala, G.; Maiorino, F.; Califano, A.; Fabiano, M.; et al. A Serum Metabolomic Signature for the Detection and Grading of Bladder Cancer. *Applied Sciences* **2021**, *11*, doi:10.3390/app11062835.

2. Troisi, J.; Tafuro, M.; Lombardi, M.; Scala, G.; Richards, S.M.; Symes, S.J.K.; Ascierto, P.A.; Delrio, P.; Tatangelo, F.; Buonerba, C.; et al. A Metabolomics-Based Screening Proposal for Colorectal Cancer. *Metabolites* **2022**, *12*, doi:10.3390/metabo12020110.

3. Troisi, J.; Raffone, A.; Travaglino, A.; Belli, G.; Belli, C.; Anand, S.; Giugliano, L.; Cavallo, P.; Scala, G.; Symes, S.; et al. Development and Validation of a Serum Metabolomic Signature for Endometrial Cancer Screening in Postmenopausal Women. *JAMA Network Open* **2020**, *3*, e2018327–e2018327, doi:10.1001/jamanetworkopen.2020.18327.

4. Troisi, J.; Sarno, L.; Landolfi, A.; Scala, G.; Martinelli, P.; Venturella, R.; Di Cello, A.; Zullo, F.; Guida, M. Metabolomic Signature of Endometrial Cancer. *J. Proteome Res.* **2018**, *17*, 804–812, doi:10.1021/acs.jproteome.7b00503.

5. Troisi, J.; Mollo, A.; Lombardi, M.; Scala, G.; Richards, S.M.; Symes, S.J.K.; Travaglino, A.; Neola, D.; de Laurentiis, U.; Insabato, L.; et al. The Metabolomic Approach for the Screening of Endometrial Cancer: Validation from a Large Cohort of Women Scheduled for Gynecological Surgery. *Biomolecules* **2022**, *12*, 1229, doi:10.3390/biom12091229.

6. Troisi, J.; Cavallo, P.; Richards, S.; Symes, S.; Colucci, A.; Sarno, L.; Landolfi, A.; Scala, G.; Adair, D.; Ciccone, C. Noninvasive Screening for Congenital Heart Defects Using a Serum Metabolomics Approach. *Prenatal Diagnosis* **2021**, *41*, 743–753.

7. Troisi, J.; Landolfi, A.; Sarno, L.; Richards, S.; Symes, S.; Adair, D.; Ciccone, C.; Scala, G.; Martinelli, P.; Guida, M. A Metabolomics-Based Approach for Non-Invasive Screening of Fetal Central Nervous System Anomalies. *Metabolomics* **2018**, *14*, 1–10.

8. Troisi, J.; Sarno, L.; Martinelli, P.; Di Carlo, C.; Landolfi, A.; Scala, G.; Rinaldi, M.; D'Alessandro, P.; Ciccone, C.; Guida, M. A Metabolomics-Based Approach for Non-Invasive Diagnosis of Chromosomal Anomalies. *Metabolomics* **2017**, *13*, 140, doi:10.1007/s11306-017-1274-z.

9. Troisi, J.; Lombardi, M.; Scala, G.; Cavallo, P.; Tayler, R.S.; Symes, S.J.K.; Richards, S.M.; Adair, D.C.; Fasano, A.; McCowan, L.M.; et al. A Screening Test Proposal for Congenital Defects Based on Maternal Serum Metabolomics Profile. *American Journal of Obstetrics and Gynecology* **2022**, S0002937822007177, doi:10.1016/j.ajog.2022.08.050.
