# Supplementary material for: Double-Weighted Bayesian Model Combination for Metabolomics Data Description and Prediction
Source: Metabolites. 2025 Mar 21;15(4):214. doi: 10.3390/metabo15040214 (PMC12029032; doi:10.3390/metabo15040214)
Supplement: Supplementary file 1 [file metabolites-15-00214-s001.zip › metabolites-3488782-supplementary/Supplementary S2.pdf]

**Supplementary 2: DW-EML single classification performance**

**Arturas Grauslys et all.**

- **Dataset CB**

Full Optimizer

| | | Estimate | Standard Error |
|---|---|---|---|
| GLM | Sensitivity | 1.00 | 0.00 |
| | Specificity | 0.91 | 0.06 |
| | Positive Likelihood ratio | 11.00 | |
| | Negative Likelihood ratio | 0.00 | |
| | Negative predictive value | 1.00 | 0.00 |
| | Positive predictive value | 0.91 | 0.06 |
| | Accuracy | 0.952 | |
| FLM | | Estimate | Standard Error |
| | Sensitivity | 0.80 | 0.09 |
| | Specificity | 0.68 | 0.10 |
| | Positive Likelihood ratio | 2.51 | |
| | Negative Likelihood ratio | 0.29 | |
| | Negative predictive value | 0.79 | 0.09 |
| | Positive predictive value | 0.70 | 0.10 |
| | Accuracy | 0.738 | |
| DL | | Estimate | Standard Error |
| | Sensitivity | 0.95 | 0.05 |
| | Specificity | 0.68 | 0.10 |
| | Positive Likelihood ratio | 2.99 | |
| | Negative Likelihood ratio | 0.07 | |
| | Negative predictive value | 0.94 | 0.06 |
| | Positive predictive value | 0.73 | 0.09 |
| | Accuracy | 0.810 | |
| DT | | Estimate | Standard Error |
| | Sensitivity | 0.80 | 0.09 |
| | Specificity | 0.55 | 0.11 |
| | Positive Likelihood ratio | 1.76 | |
| | Negative Likelihood ratio | 0.37 | |
| | Negative predictive value | 0.75 | 0.11 |
| | Positive predictive value | 0.62 | 0.10 |
| | Accuracy | 0.667 | |
| RF | | Estimate | Standard Error |
| | Sensitivity | 0.20 | 0.09 |
| | Specificity | 1.00 | 0.00 |
| | Positive Likelihood ratio | #DIV/0! | |
| | Negative Likelihood ratio | 0.80 | |
| | Negative predictive value | 0.56 | 0.08 |
| | Positive predictive value | 1.00 | 0.00 |
| | Accuracy | 0.600 | |
| GBT | | Estimate | Standard Error |
| | Sensitivity | 0.75 | 0.10 |
| | Specificity | 0.91 | 0.06 |
| | Positive Likelihood ratio | 8.25 | |
| | Negative Likelihood ratio | 0.28 | |
| | Negative predictive value | 0.80 | 0.08 |
| | Positive predictive value | 0.88 | 0.08 |
| | Accuracy | 0.833 | |
| Esemble | | Estimate | Standard Error |
| | Sensitivity | 0.95 | 0.05 |
| | Specificity | 0.91 | 0.06 |
| | Positive Likelihood ratio | 10.45 | |
| | Negative Likelihood ratio | 0.06 | |
| | Negative predictive value | 0.95 | 0.05 |
| | Positive predictive value | 0.90 | 0.06 |
| | **Accuracy** | **0.929** | |

No Feature Selection

| | | Estimate | Standard Error |
|---|---|---|---|
| GLM | Sensitivity | 0.95 | 0.05 |
| | Specificity | 0.91 | 0.06 |
| | Positive Likelihood ratio | 10.45 | |
| | Negative Likelihood ratio | 0.06 | |
| | Negative predictive value | 0.95 | 0.05 |
| | Positive predictive value | 0.90 | 0.06 |
| | Accuracy | 0.929 | |
| LR | | Estimate | Standard Error |
| | Sensitivity | 0.70 | 0.10 |
| | Specificity | 0.45 | 0.11 |
| | Positive Likelihood ratio | 1.28 | |
| | Negative Likelihood ratio | 0.66 | |
| | Negative predictive value | 0.63 | 0.12 |
| | Positive predictive value | 0.54 | 0.10 |
| | Accuracy | 0.571 | |
| FLM | | Estimate | Standard Error |
| | Sensitivity | 0.60 | 0.11 |
| | Specificity | 0.95 | 0.04 |
| | Positive Likelihood ratio | 13.20 | |
| | Negative Likelihood ratio | 0.42 | |
| | Negative predictive value | 0.72 | 0.08 |
| | Positive predictive value | 0.92 | 0.07 |
| | Accuracy | 0.786 | |
| DL | | Estimate | Standard Error |
| | Sensitivity | 0.55 | 0.11 |
| | Specificity | 1.00 | 0.00 |
| | Positive Likelihood ratio | #DIV/0! | |
| | Negative Likelihood ratio | 0.45 | |
| | Negative predictive value | 0.71 | 0.08 |
| | Positive predictive value | 1.00 | 0.00 |
| | Accuracy | 0.786 | |
| RF | | Estimate | Standard Error |
| | Sensitivity | 0.25 | 0.10 |
| | Specificity | 1.00 | 0.00 |
| | Positive Likelihood ratio | #DIV/0! | |
| | Negative Likelihood ratio | 0.75 | |
| | Negative predictive value | 0.59 | 0.08 |
| | Positive predictive value | 1.00 | 0.00 |
| | Accuracy | 0.643 | |
| GBT | | Estimate | Standard Error |
| | Sensitivity | 0.45 | 0.11 |
| | Specificity | 0.68 | 0.10 |
| | Positive Likelihood ratio | 1.41 | |
| | Negative Likelihood ratio | 0.81 | |
| | Negative predictive value | 0.58 | 0.10 |
| | Positive predictive value | 0.56 | 0.12 |
| | Accuracy | 0.571 | |
| SVM | | Estimate | Standard Error |
| | Sensitivity | 0.60 | 0.11 |
| | Specificity | 0.95 | 0.04 |
| | Positive Likelihood ratio | 13.20 | |
| | Negative Likelihood ratio | 0.42 | |
| | Negative predictive value | 0.72 | 0.08 |
| | Positive predictive value | 0.92 | 0.07 |
| | Accuracy | 0.786 | |
| Esemble | | Estimate | Standard Error |
| | Sensitivity | 0.95 | 0.05 |
| | Specificity | 0.86 | 0.07 |
| | Positive Likelihood ratio | 6.97 | |
| | Negative Likelihood ratio | 0.06 | |
| | Negative predictive value | 0.95 | 0.05 |
| | Positive predictive value | 0.86 | 0.07 |
| | **Accuracy** | **0.905** | |

| No Optimization | | | |
|---|---|---|---|
| | | Estimate | Standard Error |
| GLM | Sensitivity | 1.00 | 0.00 |
| | Specificity | 0.68 | 0.10 |
| | Positive Likelihood ratio | 3.14 | |
| | Negative Likelihood ratio | 0.00 | |
| | Negative predictive value | 1.00 | 0.00 |
| | Positive predictive value | 0.74 | 0.08 |
| | Accuracy | 0.833 | |
| | | Estimate | Standard Error |
| FLM | Sensitivity | 1.00 | 0.00 |
| | Specificity | 0.50 | 0.11 |
| | Positive Likelihood ratio | 2.00 | |
| | Negative Likelihood ratio | 0.00 | |
| | Negative predictive value | 1.00 | 0.00 |
| | Positive predictive value | 0.65 | 0.09 |
| | Accuracy | 0.738 | |
| | | Estimate | Standard Error |
| DL | Sensitivity | 0.60 | 0.11 |
| | Specificity | 1.00 | 0.00 |
| | Positive Likelihood ratio | #DIV/0! | |
| | Negative Likelihood ratio | 0.40 | |
| | Negative predictive value | 0.73 | 0.08 |
| | Positive predictive value | 1.00 | 0.00 |
| | Accuracy | 0.810 | |
| | | Estimate | Standard Error |
| GBT | Sensitivity | 0.95 | 0.05 |
| | Specificity | 0.55 | 0.11 |
| | Positive Likelihood ratio | 2.09 | |
| | Negative Likelihood ratio | 0.09 | |
| | Negative predictive value | 0.92 | 0.07 |
| | Positive predictive value | 0.66 | 0.09 |
| | Accuracy | 0.738 | |
| | | Estimate | Standard Error |
| SVM | Sensitivity | 0.80 | 0.09 |
| | Specificity | 0.68 | 0.10 |
| | Positive Likelihood ratio | 2.51 | |
| | Negative Likelihood ratio | 0.29 | |
| | Negative predictive value | 0.79 | 0.09 |
| | Positive predictive value | 0.70 | 0.10 |
| | Accuracy | 0.738 | |
| | | Estimate | Standard Error |
| Esemble | Sensitivity | 0.83 | 0.08 |
| | Specificity | 0.95 | 0.05 |
| | Positive Likelihood ratio | 15.70 | |
| | Negative Likelihood ratio | 0.18 | |
| | Negative predictive value | 0.82 | 0.08 |
| | Positive predictive value | 0.95 | 0.05 |
| | **Accuracy** | **0.881** | |

| No Feature Selection and Optimization | | | |
|---|---|---|---|
| | | Estimate | Standard Error |
| GLM | Sensitivity | 0.95 | 0.05 |
| | Specificity | 0.91 | 0.06 |
| | Positive Likelihood ratio | 10.45 | |
| | Negative Likelihood ratio | 0.06 | |
| | Negative predictive value | 0.95 | 0.05 |
| | Positive predictive value | 0.90 | 0.06 |
| | Accuracy | 0.929 | |
| | | Estimate | Standard Error |
| LR | Sensitivity | 0.70 | 0.10 |
| | Specificity | 0.45 | 0.11 |
| | Positive Likelihood ratio | 1.28 | |
| | Negative Likelihood ratio | 0.66 | |
| | Negative predictive value | 0.63 | 0.12 |
| | Positive predictive value | 0.54 | 0.10 |
| | Accuracy | 0.571 | |
| | | Estimate | Standard Error |
| FLM | Sensitivity | 1.00 | 0.00 |
| | Specificity | 0.41 | 0.10 |
| | Positive Likelihood ratio | 1.69 | |
| | Negative Likelihood ratio | 0.00 | |
| | Negative predictive value | 1.00 | 0.00 |
| | Positive predictive value | 0.61 | 0.09 |
| | Accuracy | 0.690 | |
| | | Estimate | Standard Error |
| DL | Sensitivity | 0.55 | 0.11 |
| | Specificity | 1.00 | 0.00 |
| | Positive Likelihood ratio | #DIV/0! | |
| | Negative Likelihood ratio | 0.45 | |
| | Negative predictive value | 0.71 | 0.08 |
| | Positive predictive value | 1.00 | 0.00 |
| | Accuracy | 0.786 | |
| | | Estimate | Standard Error |
| DT | Sensitivity | 0.90 | 0.07 |
| | Specificity | 0.45 | 0.11 |
| | Positive Likelihood ratio | 1.65 | |
| | Negative Likelihood ratio | 0.22 | |
| | Negative predictive value | 0.83 | 0.11 |
| | Positive predictive value | 0.60 | 0.09 |
| | Accuracy | 0.667 | |
| | | Estimate | Standard Error |
| RF | Sensitivity | 0.80 | 0.09 |
| | Specificity | 0.95 | 0.04 |
| | Positive Likelihood ratio | 17.60 | |
| | Negative Likelihood ratio | 0.21 | |
| | Negative predictive value | 0.84 | 0.07 |
| | Positive predictive value | 0.94 | 0.06 |
| | Accuracy | 0.881 | |
| | | Estimate | Standard Error |
| GBT | Sensitivity | 0.90 | 0.07 |
| | Specificity | 0.55 | 0.11 |
| | Positive Likelihood ratio | 1.98 | |
| | Negative Likelihood ratio | 0.18 | |
| | Negative predictive value | 0.86 | 0.09 |
| | Positive predictive value | 0.64 | 0.09 |
| | Accuracy | 0.714 | |
| | | Estimate | Standard Error |
| SVM | Sensitivity | 0.85 | 0.08 |
| | Specificity | 0.91 | 0.06 |
| | Positive Likelihood ratio | 9.35 | |
| | Negative Likelihood ratio | 0.17 | |
| | Negative predictive value | 0.87 | 0.07 |
| | Positive predictive value | 0.89 | 0.07 |
| | Accuracy | 0.881 | |
| | | Estimate | Standard Error |
| Esemble | Sensitivity | 0.95 | 0.05 |
| | Specificity | 0.95 | 0.04 |
| | Positive Likelihood ratio | 20.90 | |
| | Negative Likelihood ratio | 0.05 | |
| | Negative predictive value | 0.95 | 0.04 |
| | Positive predictive value | 0.95 | 0.05 |
| | **Accuracy** | **0.952** | |

- **Dataset CV**

| Full Optimizer | | | |
|---|---|---|---|
| | | Estimate | Standard Error |
| NB | Sensitivity | 0.80 | 0.09 |
| | Specificity | 0.68 | 0.10 |
| | Positive Likelihood ratio | 2.51 | |
| | Negative Likelihood ratio | 0.29 | |
| | Negative predictive value | 0.79 | 0.09 |
| | Positive predictive value | 0.70 | 0.10 |
| | Accuracy | 0.738 | |
| | | Estimate | Standard Error |
| GLM | Sensitivity | 0.75 | 0.10 |
| | Specificity | 0.91 | 0.06 |
| | Positive Likelihood ratio | 8.25 | |
| | Negative Likelihood ratio | 0.28 | |
| | Negative predictive value | 0.80 | 0.08 |
| | Positive predictive value | 0.88 | 0.08 |
| | Accuracy | 0.833 | |
| | | Estimate | Standard Error |
| FLM | Sensitivity | 0.75 | 0.10 |
| | Specificity | 0.95 | 0.04 |
| | Positive Likelihood ratio | 16.50 | |
| | Negative Likelihood ratio | 0.26 | |
| | Negative predictive value | 0.81 | 0.08 |
| | Positive predictive value | 0.94 | 0.06 |
| | Accuracy | 0.857 | |
| | | Estimate | Standard Error |
| DL | Sensitivity | 0.60 | 0.11 |
| | Specificity | 0.95 | 0.04 |
| | Positive Likelihood ratio | 13.20 | |
| | Negative Likelihood ratio | 0.42 | |
| | Negative predictive value | 0.72 | 0.08 |
| | Positive predictive value | 0.92 | 0.07 |
| | Accuracy | 0.786 | |
| | | Estimate | Standard Error |
| DT | Sensitivity | 0.45 | 0.11 |
| | Specificity | 0.95 | 0.04 |
| | Positive Likelihood ratio | 9.90 | |
| | Negative Likelihood ratio | 0.58 | |
| | Negative predictive value | 0.66 | 0.08 |
| | Positive predictive value | 0.90 | 0.09 |
| | Accuracy | 0.714 | |
| | | Estimate | Standard Error |
| RF | Sensitivity | 0.35 | 0.11 |
| | Specificity | 0.91 | 0.06 |
| | Positive Likelihood ratio | 3.85 | |
| | Negative Likelihood ratio | 0.72 | |
| | Negative predictive value | 0.61 | 0.09 |
| | Positive predictive value | 0.78 | 0.14 |
| | Accuracy | 0.643 | |
| | | Estimate | Standard Error |
| GBT | Sensitivity | 0.50 | 0.11 |
| | Specificity | 0.73 | 0.09 |
| | Positive Likelihood ratio | 1.83 | |
| | Negative Likelihood ratio | 0.69 | |
| | Negative predictive value | 0.62 | 0.10 |
| | Positive predictive value | 0.63 | 0.12 |
| | Accuracy | 0.619 | |
| | | Estimate | Standard Error |
| SVM | Sensitivity | 0.70 | 0.10 |
| | Specificity | 0.77 | 0.09 |
| | Positive Likelihood ratio | 3.08 | |
| | Negative Likelihood ratio | 0.39 | |
| | Negative predictive value | 0.74 | 0.09 |
| | Positive predictive value | 0.74 | 0.10 |
| | Accuracy | 0.738 | |
| | | Estimate | Standard Error |
| Esemble | Sensitivity | 0.85 | 0.08 |
| | Specificity | 0.91 | 0.06 |
| | Positive Likelihood ratio | 9.35 | |
| | Negative Likelihood ratio | 0.17 | |
| | Negative predictive value | 0.87 | 0.07 |
| | Positive predictive value | 0.89 | 0.07 |
| | **Accuracy** | **0.881** | |

| No Feature Selection | | | |
|---|---|---|---|
| | | Estimate | Standard Error |
| NB | Sensitivity | 0.60 | 0.11 |
| | Specificity | 0.82 | 0.08 |
| | Positive Likelihood ratio | 3.30 | |
| | Negative Likelihood ratio | 0.49 | |
| | Negative predictive value | 0.69 | 0.09 |
| | Positive predictive value | 0.75 | 0.11 |
| | Accuracy | 0.714 | |
| | | Estimate | Standard Error |
| GLM | Sensitivity | 0.45 | 0.11 |
| | Specificity | 1.00 | 0.00 |
| | Positive Likelihood ratio | #DIV/0! | |
| | Negative Likelihood ratio | 0.55 | |
| | Negative predictive value | 0.67 | 0.08 |
| | Positive predictive value | 1.00 | 0.00 |
| | Accuracy | 0.738 | |
| | | Estimate | Standard Error |
| FLM | Sensitivity | 0.45 | 0.11 |
| | Specificity | 1.00 | 0.00 |
| | Positive Likelihood ratio | #DIV/0! | |
| | Negative Likelihood ratio | 0.55 | |
| | Negative predictive value | 0.67 | 0.08 |
| | Positive predictive value | 1.00 | 0.00 |
| | Accuracy | 0.738 | |
| | | Estimate | Standard Error |
| DL | Sensitivity | 0.75 | 0.10 |
| | Specificity | 0.95 | 0.04 |
| | Positive Likelihood ratio | 16.50 | |
| | Negative Likelihood ratio | 0.26 | |
| | Negative predictive value | 0.81 | 0.08 |
| | Positive predictive value | 0.94 | 0.06 |
| | Accuracy | 0.857 | |
| | | Estimate | Standard Error |
| DT | Sensitivity | 0.45 | 0.11 |
| | Specificity | 0.95 | 0.04 |
| | Positive Likelihood ratio | 9.90 | |
| | Negative Likelihood ratio | 0.58 | |
| | Negative predictive value | 0.66 | 0.08 |
| | Positive predictive value | 0.90 | 0.09 |
| | Accuracy | 0.714 | |
| | | Estimate | Standard Error |
| RF | Sensitivity | 0.60 | 0.11 |
| | Specificity | 0.91 | 0.06 |
| | Positive Likelihood ratio | 6.60 | |
| | Negative Likelihood ratio | 0.44 | |
| | Negative predictive value | 0.71 | 0.09 |
| | Positive predictive value | 0.86 | 0.09 |
| | Accuracy | 0.762 | |
| | | Estimate | Standard Error |
| GBT | Sensitivity | 0.25 | 0.10 |
| | Specificity | 1.00 | 0.00 |
| | Positive Likelihood ratio | #DIV/0! | |
| | Negative Likelihood ratio | 0.75 | |
| | Negative predictive value | 0.59 | 0.08 |
| | Positive predictive value | 1.00 | 0.00 |
| | Accuracy | 0.643 | |
| | | Estimate | Standard Error |
| SVM | Sensitivity | 0.70 | 0.10 |
| | Specificity | 0.77 | 0.09 |
| | Positive Likelihood ratio | 3.08 | |
| | Negative Likelihood ratio | 0.39 | |
| | Negative predictive value | 0.74 | 0.09 |
| | Positive predictive value | 0.74 | 0.10 |
| | Accuracy | 0.738 | |
| | | Estimate | Standard Error |
| Esemble | Sensitivity | 0.75 | 0.10 |
| | Specificity | 0.86 | 0.07 |
| | Positive Likelihood ratio | 5.50 | |
| | Negative Likelihood ratio | 0.29 | |
| | Negative predictive value | 0.79 | 0.08 |
| | Positive predictive value | 0.83 | 0.09 |
| | **Accuracy** | **0.810** | |

| No Optimization | | | |
|---|---|---|---|
| | | Estimate | Standard Error |
| NB | Sensitivity | 0.60 | 0.11 |
| | Specificity | 0.82 | 0.08 |
| | Positive Likelihood ratio | 3.30 | |
| | Negative Likelihood ratio | 0.49 | |
| | Negative predictive value | 0.69 | 0.09 |
| | Positive predictive value | 0.75 | 0.11 |
| | Accuracy | 0.714 | |
| | | Estimate | Standard Error |
| GLM | Sensitivity | 0.50 | 0.11 |
| | Specificity | 1.00 | 0.00 |
| | Positive Likelihood ratio | #DIV/0! | |
| | Negative Likelihood ratio | 0.50 | |
| | Negative predictive value | 0.69 | 0.08 |
| | Positive predictive value | 1.00 | 0.00 |
| | Accuracy | 0.762 | |
| | | Estimate | Standard Error |
| FLM | Sensitivity | 0.90 | 0.07 |
| | Specificity | 0.45 | 0.11 |
| | Positive Likelihood ratio | 1.65 | |
| | Negative Likelihood ratio | 0.22 | |
| | Negative predictive value | 0.83 | 0.11 |
| | Positive predictive value | 0.60 | 0.09 |
| | Accuracy | 0.667 | |
| | | Estimate | Standard Error |
| DL | Sensitivity | 0.60 | 0.11 |
| | Specificity | 0.95 | 0.04 |
| | Positive Likelihood ratio | 13.20 | |
| | Negative Likelihood ratio | 0.42 | |
| | Negative predictive value | 0.72 | 0.08 |
| | Positive predictive value | 0.92 | 0.07 |
| | Accuracy | 0.786 | |
| | | Estimate | Standard Error |
| DT | Sensitivity | 0.50 | 0.11 |
| | Specificity | 0.91 | 0.06 |
| | Positive Likelihood ratio | 5.50 | |
| | Negative Likelihood ratio | 0.55 | |
| | Negative predictive value | 0.67 | 0.09 |
| | Positive predictive value | 0.83 | 0.11 |
| | Accuracy | 0.714 | |
| | | Estimate | Standard Error |
| RF | Sensitivity | 0.70 | 0.10 |
| | Specificity | 0.86 | 0.07 |
| | Positive Likelihood ratio | 5.13 | |
| | Negative Likelihood ratio | 0.35 | |
| | Negative predictive value | 0.76 | 0.09 |
| | Positive predictive value | 0.82 | 0.09 |
| | Accuracy | 0.786 | |
| | | Estimate | Standard Error |
| GBT | Sensitivity | 0.40 | 0.11 |
| | Specificity | 1.00 | 0.00 |
| | Positive Likelihood ratio | #DIV/0! | |
| | Negative Likelihood ratio | 0.60 | |
| | Negative predictive value | 0.65 | 0.08 |
| | Positive predictive value | 1.00 | 0.00 |
| | Accuracy | 0.714 | |
| | | Estimate | Standard Error |
| Esemble | Sensitivity | 0.70 | 0.10 |
| | Specificity | 1.00 | 0.00 |
| | Positive Likelihood ratio | #DIV/0! | |
| | Negative Likelihood ratio | 0.30 | |
| | Negative predictive value | 0.79 | 0.08 |
| | Positive predictive value | 1.00 | 0.00 |
| | **Accuracy** | **0.857** | |

| No Feature Selection and Optimization | | | |
|---|---|---|---|
| | | Estimate | Standard Error |
| NB | Sensitivity | 0.60 | 0.11 |
| | Specificity | 0.82 | 0.08 |
| | Positive Likelihood ratio | 3.30 | |
| | Negative Likelihood ratio | 0.49 | |
| | Negative predictive value | 0.69 | 0.09 |
| | Positive predictive value | 0.75 | 0.11 |
| | Accuracy | 0.714 | |
| | | Estimate | Standard Error |
| GLM | Sensitivity | 0.55 | 0.11 |
| | Specificity | 1.00 | 0.00 |
| | Positive Likelihood ratio | #DIV/0! | |
| | Negative Likelihood ratio | 0.45 | |
| | Negative predictive value | 0.71 | 0.08 |
| | Positive predictive value | 1.00 | 0.00 |
| | Accuracy | 0.786 | |
| | | Estimate | Standard Error |
| FLM | Sensitivity | 0.45 | 0.11 |
| | Specificity | 1.00 | 0.00 |
| | Positive Likelihood ratio | #DIV/0! | |
| | Negative Likelihood ratio | 0.55 | |
| | Negative predictive value | 0.67 | 0.08 |
| | Positive predictive value | 1.00 | 0.00 |
| | Accuracy | 0.738 | |
| | | Estimate | Standard Error |
| DL | Sensitivity | 0.75 | 0.10 |
| | Specificity | 0.95 | 0.04 |
| | Positive Likelihood ratio | 16.50 | |
| | Negative Likelihood ratio | 0.26 | |
| | Negative predictive value | 0.81 | 0.08 |
| | Positive predictive value | 0.94 | 0.06 |
| | Accuracy | 0.857 | |
| | | Estimate | Standard Error |
| DT | Sensitivity | 0.50 | 0.11 |
| | Specificity | 0.91 | 0.06 |
| | Positive Likelihood ratio | 5.50 | |
| | Negative Likelihood ratio | 0.55 | |
| | Negative predictive value | 0.67 | 0.09 |
| | Positive predictive value | 0.83 | 0.11 |
| | Accuracy | 0.714 | |
| | | Estimate | Standard Error |
| RF | Sensitivity | 0.70 | 0.10 |
| | Specificity | 0.73 | 0.09 |
| | Positive Likelihood ratio | 2.57 | |
| | Negative Likelihood ratio | 0.41 | |
| | Negative predictive value | 0.73 | 0.09 |
| | Positive predictive value | 0.70 | 0.10 |
| | Accuracy | 0.714 | |
| | | Estimate | Standard Error |
| GBT | Sensitivity | 0.25 | 0.10 |
| | Specificity | 1.00 | 0.00 |
| | Positive Likelihood ratio | #DIV/0! | |
| | Negative Likelihood ratio | 0.75 | |
| | Negative predictive value | 0.59 | 0.08 |
| | Positive predictive value | 1.00 | 0.00 |
| | Accuracy | 0.643 | |
| | | Estimate | Standard Error |
| SVM | Sensitivity | 0.75 | 0.10 |
| | Specificity | 0.86 | 0.07 |
| | Positive Likelihood ratio | 5.50 | |
| | Negative Likelihood ratio | 0.29 | |
| | Negative predictive value | 0.79 | 0.08 |
| | Positive predictive value | 0.83 | 0.09 |
| | Accuracy | 0.810 | |
| | | Estimate | Standard Error |
| Esemble | Sensitivity | 0.70 | 0.10 |
| | Specificity | 0.95 | 0.04 |
| | Positive Likelihood ratio | 15.40 | |
| | Negative Likelihood ratio | 0.31 | |
| | Negative predictive value | 0.78 | 0.08 |
| | Positive predictive value | 0.93 | 0.06 |
| | **Accuracy** | **0.833** | |

## - Dataset CB-CV

| Full Optimizer | | | |
|---|---|---|---|
| NB | | Estimate | Standard Error |
| | Sensitivity | 0.74 | 0.10 |
| | Specificity | 0.95 | 0.05 |
| | Positive Likelihood ratio | 15.47 | |
| | Negative Likelihood ratio | 0.28 | |
| | Negative predictive value | 0.80 | 0.08 |
| | Positive predictive value | 0.93 | 0.06 |
| | Accuracy | 0.850 | |
| GLM | | Estimate | Standard Error |
| | Sensitivity | 1.00 | 0.00 |
| | Specificity | 0.72 | 0.08 |
| | Positive Likelihood ratio | 3.63 | |
| | Negative Likelihood ratio | 0.00 | |
| | Negative predictive value | 1.00 | 0.00 |
| | Positive predictive value | 0.58 | 0.11 |
| | Accuracy | 0.800 | |
| FLM | | Estimate | Standard Error |
| | Sensitivity | 1.00 | 0.00 |
| | Specificity | 0.91 | 0.06 |
| | Positive Likelihood ratio | 11.50 | |
| | Negative Likelihood ratio | 0.00 | |
| | Negative predictive value | 1.00 | 0.00 |
| | Positive predictive value | 0.89 | 0.07 |
| | Accuracy | 0.950 | |
| DL | | Estimate | Standard Error |
| | Sensitivity | 0.84 | 0.08 |
| | Specificity | 0.76 | 0.09 |
| | Positive Likelihood ratio | 3.54 | |
| | Negative Likelihood ratio | 0.21 | |
| | Negative predictive value | 0.84 | 0.08 |
| | Positive predictive value | 0.76 | 0.09 |
| | Accuracy | 0.800 | |
| DT | | Estimate | Standard Error |
| | Sensitivity | 0.79 | 0.09 |
| | Specificity | 0.71 | 0.10 |
| | Positive Likelihood ratio | 2.76 | |
| | Negative Likelihood ratio | 0.29 | |
| | Negative predictive value | 0.79 | 0.09 |
| | Positive predictive value | 0.71 | 0.10 |
| | Accuracy | 0.750 | |
| RF | | Estimate | Standard Error |
| | Sensitivity | 0.95 | 0.05 |
| | Specificity | 0.57 | 0.11 |
| | Positive Likelihood ratio | 2.21 | |
| | Negative Likelihood ratio | 0.09 | |
| | Negative predictive value | 0.92 | 0.07 |
| | Positive predictive value | 0.67 | 0.09 |
| | Accuracy | 0.750 | |
| GBT | | Estimate | Standard Error |
| | Sensitivity | 0.63 | 0.11 |
| | Specificity | 0.90 | 0.06 |
| | Positive Likelihood ratio | 6.63 | |
| | Negative Likelihood ratio | 0.41 | |
| | Negative predictive value | 0.73 | 0.09 |
| | Positive predictive value | 0.86 | 0.09 |
| | Accuracy | 0.775 | |
| SVM | | Estimate | Standard Error |
| | Sensitivity | 0.89 | 0.07 |
| | Specificity | 0.81 | 0.09 |
| | Positive Likelihood ratio | 4.70 | |
| | Negative Likelihood ratio | 0.13 | |
| | Negative predictive value | 0.89 | 0.07 |
| | Positive predictive value | 0.81 | 0.09 |
| | Accuracy | 0.850 | |
| Esemble | | Estimate | Standard Error |
| | Sensitivity | 1.00 | 0.00 |
| | Specificity | 0.86 | 0.08 |
| | Positive Likelihood ratio | 7.00 | |
| | Negative Likelihood ratio | 0.00 | |
| | Negative predictive value | 1.00 | 0.00 |
| | Positive predictive value | 0.86 | 0.07 |
| | **Accuracy** | **0.925** | |

| No Feature Selection | | | |
|---|---|---|---|
| NB | | Estimate | Standard Error |
| | Sensitivity | 0.89 | 0.07 |
| | Specificity | 0.52 | 0.11 |
| | Positive Likelihood ratio | 1.88 | |
| | Negative Likelihood ratio | 0.20 | |
| | Negative predictive value | 0.85 | 0.10 |
| | Positive predictive value | 0.63 | 0.09 |
| | Accuracy | 0.700 | |
| GLM | | Estimate | Standard Error |
| | Sensitivity | 0.89 | 0.07 |
| | Specificity | 0.95 | 0.05 |
| | Positive Likelihood ratio | 18.79 | |
| | Negative Likelihood ratio | 0.11 | |
| | Negative predictive value | 0.91 | 0.06 |
| | Positive predictive value | 0.94 | 0.05 |
| | Accuracy | 0.925 | |
| FLM | | Estimate | Standard Error |
| | Sensitivity | 0.79 | 0.09 |
| | Specificity | 0.95 | 0.05 |
| | Positive Likelihood ratio | 16.58 | |
| | Negative Likelihood ratio | 0.22 | |
| | Negative predictive value | 0.83 | 0.08 |
| | Positive predictive value | 0.94 | 0.06 |
| | Accuracy | 0.875 | |
| DL | | Estimate | Standard Error |
| | Sensitivity | 0.89 | 0.07 |
| | Specificity | 0.86 | 0.08 |
| | Positive Likelihood ratio | 6.26 | |
| | Negative Likelihood ratio | 0.12 | |
| | Negative predictive value | 0.90 | 0.07 |
| | Positive predictive value | 0.85 | 0.08 |
| | Accuracy | 0.875 | |
| DT | | Estimate | Standard Error |
| | Sensitivity | 0.84 | 0.08 |
| | Specificity | 0.76 | 0.09 |
| | Positive Likelihood ratio | 3.54 | |
| | Negative Likelihood ratio | 0.21 | |
| | Negative predictive value | 0.84 | 0.08 |
| | Positive predictive value | 0.76 | 0.09 |
| | Accuracy | 0.800 | |
| RF | | Estimate | Standard Error |
| | Sensitivity | 0.95 | 0.05 |
| | Specificity | 0.48 | 0.11 |
| | Positive Likelihood ratio | 1.81 | |
| | Negative Likelihood ratio | 0.11 | |
| | Negative predictive value | 0.91 | 0.09 |
| | Positive predictive value | 0.62 | 0.09 |
| | Accuracy | 0.700 | |
| GBT | | Estimate | Standard Error |
| | Sensitivity | 0.68 | 0.11 |
| | Specificity | 0.90 | 0.06 |
| | Positive Likelihood ratio | 7.18 | |
| | Negative Likelihood ratio | 0.35 | |
| | Negative predictive value | 0.76 | 0.09 |
| | Positive predictive value | 0.87 | 0.09 |
| | Accuracy | 0.800 | |
| SVM | | Estimate | Standard Error |
| | Sensitivity | 0.84 | 0.08 |
| | Specificity | 0.76 | 0.09 |
| | Positive Likelihood ratio | 3.54 | |
| | Negative Likelihood ratio | 0.21 | |
| | Negative predictive value | 0.84 | 0.08 |
| | Positive predictive value | 0.76 | 0.09 |
| | Accuracy | 0.800 | |
| Esemble | | Estimate | Standard Error |
| | Sensitivity | 1.00 | 0.00 |
| | Specificity | 0.81 | 0.09 |
| | Positive Likelihood ratio | 5.25 | |
| | Negative Likelihood ratio | 0.00 | |
| | Negative predictive value | 1.00 | 0.00 |
| | Positive predictive value | 0.83 | 0.08 |
| | **Accuracy** | **0.900** | |

| No Optimization | | | |
|---|---|---|---|
| NB | | Estimate | Standard Error |
| | Sensitivity | 0.74 | 0.10 |
| | Specificity | 0.95 | 0.05 |
| | Positive Likelihood ratio | 15.47 | |
| | Negative Likelihood ratio | 0.28 | |
| | Negative predictive value | 0.80 | 0.08 |
| | Positive predictive value | 0.93 | 0.06 |
| | Accuracy | 0.850 | |
| GLM | | Estimate | Standard Error |
| | Sensitivity | 0.84 | 0.08 |
| | Specificity | 0.90 | 0.06 |
| | Positive Likelihood ratio | 8.84 | |
| | Negative Likelihood ratio | 0.17 | |
| | Negative predictive value | 0.86 | 0.07 |
| | Positive predictive value | 0.89 | 0.07 |
| | Accuracy | 0.875 | |
| FLM | | Estimate | Standard Error |
| | Sensitivity | 1.00 | 0.00 |
| | Specificity | 0.81 | 0.09 |
| | Positive Likelihood ratio | 5.25 | |
| | Negative Likelihood ratio | 0.00 | |
| | Negative predictive value | 1.00 | 0.00 |
| | Positive predictive value | 0.83 | 0.08 |
| | Accuracy | 0.900 | |
| DL | | Estimate | Standard Error |
| | Sensitivity | 0.79 | 0.09 |
| | Specificity | 0.86 | 0.08 |
| | Positive Likelihood ratio | 5.53 | |
| | Negative Likelihood ratio | 0.25 | |
| | Negative predictive value | 0.82 | 0.08 |
| | Positive predictive value | 0.83 | 0.09 |
| | Accuracy | 0.825 | |
| GBT | | Estimate | Standard Error |
| | Sensitivity | 0.74 | 0.10 |
| | Specificity | 0.86 | 0.08 |
| | Positive Likelihood ratio | 5.16 | |
| | Negative Likelihood ratio | 0.31 | |
| | Negative predictive value | 0.78 | 0.09 |
| | Positive predictive value | 0.82 | 0.09 |
| | Accuracy | 0.800 | |
| SVM | | Estimate | Standard Error |
| | Sensitivity | 0.84 | 0.08 |
| | Specificity | 0.76 | 0.09 |
| | Positive Likelihood ratio | 3.54 | |
| | Negative Likelihood ratio | 0.21 | |
| | Negative predictive value | 0.84 | 0.08 |
| | Positive predictive value | 0.76 | 0.09 |
| | Accuracy | 0.800 | |
| Esemble | | Estimate | Standard Error |
| | Sensitivity | 1.00 | 0.00 |
| | Specificity | 0.81 | 0.09 |
| | Positive Likelihood ratio | 5.25 | |
| | Negative Likelihood ratio | 0.00 | |
| | Negative predictive value | 1.00 | 0.00 |
| | Positive predictive value | 0.83 | 0.08 |
| | **Accuracy** | **0.900** | |

| No Feature Selection and Optimization | | | |
|---|---|---|---|
| NB | | Estimate | Standard Error |
| | Sensitivity | 0.89 | 0.07 |
| | Specificity | 0.52 | 0.11 |
| | Positive Likelihood ratio | 1.88 | |
| | Negative Likelihood ratio | 0.20 | |
| | Negative predictive value | 0.85 | 0.10 |
| | Positive predictive value | 0.63 | 0.09 |
| | Accuracy | 0.700 | |
| GLM | | Estimate | Standard Error |
| | Sensitivity | 0.84 | 0.08 |
| | Specificity | 0.95 | 0.05 |
| | Positive Likelihood ratio | 17.68 | |
| | Negative Likelihood ratio | 0.17 | |
| | Negative predictive value | 0.87 | 0.07 |
| | Positive predictive value | 0.94 | 0.06 |
| | Accuracy | 0.900 | |
| FLM | | Estimate | Standard Error |
| | Sensitivity | 0.89 | 0.07 |
| | Specificity | 0.86 | 0.08 |
| | Positive Likelihood ratio | 6.26 | |
| | Negative Likelihood ratio | 0.12 | |
| | Negative predictive value | 0.90 | 0.07 |
| | Positive predictive value | 0.85 | 0.08 |
| | Accuracy | 0.875 | |
| DL | | Estimate | Standard Error |
| | Sensitivity | 0.89 | 0.07 |
| | Specificity | 0.86 | 0.08 |
| | Positive Likelihood ratio | 6.26 | |
| | Negative Likelihood ratio | 0.12 | |
| | Negative predictive value | 0.90 | 0.07 |
| | Positive predictive value | 0.85 | 0.08 |
| | Accuracy | 0.875 | |
| DT | | Estimate | Standard Error |
| | Sensitivity | 0.74 | 0.10 |
| | Specificity | 0.81 | 0.09 |
| | Positive Likelihood ratio | 3.87 | |
| | Negative Likelihood ratio | 0.33 | |
| | Negative predictive value | 0.77 | 0.09 |
| | Positive predictive value | 0.78 | 0.10 |
| | Accuracy | 0.775 | |
| RF | | Estimate | Standard Error |
| | Sensitivity | 1.00 | 0.00 |
| | Specificity | 0.52 | 0.11 |
| | Positive Likelihood ratio | 2.10 | |
| | Negative Likelihood ratio | 0.00 | |
| | Negative predictive value | 1.00 | 0.00 |
| | Positive predictive value | 0.66 | 0.09 |
| | Accuracy | 0.750 | |
| GBT | | Estimate | Standard Error |
| | Sensitivity | 0.63 | 0.11 |
| | Specificity | 0.90 | 0.06 |
| | Positive Likelihood ratio | 6.63 | |
| | Negative Likelihood ratio | 0.41 | |
| | Negative predictive value | 0.73 | 0.09 |
| | Positive predictive value | 0.86 | 0.09 |
| | Accuracy | 0.775 | |
| SVM | | Estimate | Standard Error |
| | Sensitivity | 0.89 | 0.07 |
| | Specificity | 0.90 | 0.06 |
| | Positive Likelihood ratio | 9.39 | |
| | Negative Likelihood ratio | 0.12 | |
| | Negative predictive value | 0.90 | 0.06 |
| | Positive predictive value | 0.89 | 0.07 |
| | Accuracy | 0.900 | |
| Esemble | | Estimate | Standard Error |
| | Sensitivity | 0.95 | 0.05 |
| | Specificity | 0.90 | 0.06 |
| | Positive Likelihood ratio | 9.95 | |
| | Negative Likelihood ratio | 0.06 | |
| | Negative predictive value | 0.95 | 0.05 |
| | Positive predictive value | 0.90 | 0.07 |
| | **Accuracy** | **0.925** | |

# Elim Nasstrom et all.

## - Dataset ST

| Full Optimizer | | | |
|---|---|---|---|
| | | Estimate | Standard Error |
| NB | Sensitivity | 0.73 | 0.09 |
| | Specificity | 0.95 | 0.05 |
| | Positive Likelihood ratio | 14.55 | |
| | Negative Likelihood ratio | 0.29 | |
| | Negative predictive value | 0.76 | 0.09 |
| | Positive predictive value | 0.94 | 0.06 |
| | Accuracy | 0.833 | |
| | | Estimate | Standard Error |
| GLM | Sensitivity | 0.90 | 0.07 |
| | Specificity | 1.00 | 0.00 |
| | Positive Likelihood ratio | #DIV/0! | |
| | Negative Likelihood ratio | 0.10 | |
| | Negative predictive value | 0.91 | 0.06 |
| | Positive predictive value | 1.00 | 0.00 |
| | Accuracy | 0.950 | |
| | | Estimate | Standard Error |
| FLM | Sensitivity | 0.95 | 0.05 |
| | Specificity | 0.90 | 0.07 |
| | Positive Likelihood ratio | 9.50 | |
| | Negative Likelihood ratio | 0.06 | |
| | Negative predictive value | 0.95 | 0.05 |
| | Positive predictive value | 0.90 | 0.06 |
| | Accuracy | 0.925 | |
| | | Estimate | Standard Error |
| DL | Sensitivity | 1.00 | 0.00 |
| | Specificity | 0.75 | 0.10 |
| | Positive Likelihood ratio | 4.00 | |
| | Negative Likelihood ratio | 0.00 | |
| | Negative predictive value | 1.00 | 0.00 |
| | Positive predictive value | 0.80 | 0.08 |
| | Accuracy | 0.875 | |
| | | Estimate | Standard Error |
| DT | Sensitivity | 0.71 | 0.09 |
| | Specificity | 0.95 | 0.05 |
| | Positive Likelihood ratio | 14.17 | |
| | Negative Likelihood ratio | 0.31 | |
| | Negative predictive value | 0.73 | 0.09 |
| | Positive predictive value | 0.94 | 0.05 |
| | Accuracy | 0.818 | |
| | | Estimate | Standard Error |
| RF | Sensitivity | 1.00 | 0.00 |
| | Specificity | 0.95 | 0.05 |
| | Positive Likelihood ratio | 20.00 | |
| | Negative Likelihood ratio | 0.00 | |
| | Negative predictive value | 1.00 | 0.00 |
| | Positive predictive value | 0.95 | 0.05 |
| | Accuracy | 0.975 | |
| | | Estimate | Standard Error |
| GBT | Sensitivity | 0.95 | 0.05 |
| | Specificity | 0.90 | 0.07 |
| | Positive Likelihood ratio | 9.50 | |
| | Negative Likelihood ratio | 0.06 | |
| | Negative predictive value | 0.95 | 0.05 |
| | Positive predictive value | 0.90 | 0.06 |
| | Accuracy | 0.925 | |
| | | Estimate | Standard Error |
| SVM | Sensitivity | 1.00 | 0.00 |
| | Specificity | 1.00 | 0.00 |
| | Positive Likelihood ratio | #DIV/0! | |
| | Negative Likelihood ratio | 0.00 | |
| | Negative predictive value | 1.00 | 0.00 |
| | Positive predictive value | 1.00 | 0.00 |
| | Accuracy | 1.000 | |
| | | Estimate | Standard Error |
| Esemble | Sensitivity | 1.00 | 0.00 |
| | Specificity | 1.00 | 0.00 |
| | Positive Likelihood ratio | #DIV/0! | |
| | Negative Likelihood ratio | 0.00 | |
| | Negative predictive value | 1.00 | 0.00 |
| | Positive predictive value | 1.00 | 0.00 |
| | **Accuracy** | **1.000** | |

| No Feature Selection | | | |
|---|---|---|---|
| | | Estimate | Standard Error |
| NB | Sensitivity | 1.00 | 0.00 |
| | Specificity | 1.00 | 0.00 |
| | Positive Likelihood ratio | #DIV/0! | |
| | Negative Likelihood ratio | 0.00 | |
| | Negative predictive value | 1.00 | 0.00 |
| | Positive predictive value | 1.00 | 0.00 |
| | Accuracy | 1.000 | |
| | | Estimate | Standard Error |
| GLM | Sensitivity | 1.00 | 0.00 |
| | Specificity | 1.00 | 0.00 |
| | Positive Likelihood ratio | #DIV/0! | |
| | Negative Likelihood ratio | 0.00 | |
| | Negative predictive value | 1.00 | 0.00 |
| | Positive predictive value | 1.00 | 0.00 |
| | Accuracy | 1.000 | |
| | | Estimate | Standard Error |
| LR | Sensitivity | 0.95 | 0.05 |
| | Specificity | 0.95 | 0.05 |
| | Positive Likelihood ratio | 19.00 | |
| | Negative Likelihood ratio | 0.05 | |
| | Negative predictive value | 0.95 | 0.05 |
| | Positive predictive value | 0.95 | 0.05 |
| | Accuracy | 0.950 | |
| | | Estimate | Standard Error |
| FLM | Sensitivity | 1.00 | 0.00 |
| | Specificity | 1.00 | 0.00 |
| | Positive Likelihood ratio | #DIV/0! | |
| | Negative Likelihood ratio | 0.00 | |
| | Negative predictive value | 1.00 | 0.00 |
| | Positive predictive value | 1.00 | 0.00 |
| | Accuracy | 1.000 | |
| | | Estimate | Standard Error |
| DL | Sensitivity | 0.95 | 0.05 |
| | Specificity | 0.95 | 0.05 |
| | Positive Likelihood ratio | 19.00 | |
| | Negative Likelihood ratio | 0.05 | |
| | Negative predictive value | 0.95 | 0.05 |
| | Positive predictive value | 0.95 | 0.05 |
| | Accuracy | 0.950 | |
| | | Estimate | Standard Error |
| DT | Sensitivity | 0.85 | 0.08 |
| | Specificity | 1.00 | 0.00 |
| | Positive Likelihood ratio | #DIV/0! | |
| | Negative Likelihood ratio | 0.15 | |
| | Negative predictive value | 0.87 | 0.07 |
| | Positive predictive value | 1.00 | 0.00 |
| | Accuracy | 0.925 | |
| | | Estimate | Standard Error |
| RF | Sensitivity | 1.00 | 0.00 |
| | Specificity | 0.90 | 0.07 |
| | Positive Likelihood ratio | 10.00 | |
| | Negative Likelihood ratio | 0.00 | |
| | Negative predictive value | 1.00 | 0.00 |
| | Positive predictive value | 0.91 | 0.06 |
| | Accuracy | 0.950 | |
| | | Estimate | Standard Error |
| GBT | Sensitivity | 0.85 | 0.08 |
| | Specificity | 0.95 | 0.05 |
| | Positive Likelihood ratio | 17.00 | |
| | Negative Likelihood ratio | 0.16 | |
| | Negative predictive value | 0.86 | 0.07 |
| | Positive predictive value | 0.94 | 0.05 |
| | Accuracy | 0.900 | |
| | | Estimate | Standard Error |
| SVM | Sensitivity | 1.00 | 0.00 |
| | Specificity | 0.95 | 0.05 |
| | Positive Likelihood ratio | 20.00 | |
| | Negative Likelihood ratio | 0.00 | |
| | Negative predictive value | 1.00 | 0.00 |
| | Positive predictive value | 0.95 | 0.05 |
| | Accuracy | 0.975 | |
| | | Estimate | Standard Error |
| Esemble | Sensitivity | 1.00 | 0.00 |
| | Specificity | 1.00 | 0.00 |
| | Positive Likelihood ratio | #DIV/0! | |
| | Negative Likelihood ratio | 0.00 | |
| | Negative predictive value | 1.00 | 0.00 |
| | Positive predictive value | 1.00 | 0.00 |
| | **Accuracy** | **1.000** | |

| No Optimization | | | |
|---|---|---|---|
| | | Estimate | Standard Error |
| NB | Sensitivity | 0.80 | 0.09 |
| | Specificity | 0.95 | 0.05 |
| | Positive Likelihood ratio | 16.00 | |
| | Negative Likelihood ratio | 0.21 | |
| | Negative predictive value | 0.83 | 0.08 |
| | Positive predictive value | 0.94 | 0.06 |
| | Accuracy | 0.875 | |
| GLM | | Estimate | Standard Error |
| | Sensitivity | 0.90 | 0.07 |
| | Specificity | 1.00 | 0.00 |
| | Positive Likelihood ratio | #DIV/0! | |
| | Negative Likelihood ratio | 0.10 | |
| | Negative predictive value | 0.91 | 0.06 |
| | Positive predictive value | 1.00 | 0.00 |
| | Accuracy | 0.950 | |
| LR | | Estimate | Standard Error |
| | Sensitivity | 1.00 | 0.00 |
| | Specificity | 0.80 | 0.09 |
| | Positive Likelihood ratio | 5.00 | |
| | Negative Likelihood ratio | 0.00 | |
| | Negative predictive value | 1.00 | 0.00 |
| | Positive predictive value | 0.83 | 0.08 |
| | Accuracy | 0.900 | |
| DL | | Estimate | Standard Error |
| | Sensitivity | 1.00 | 0.00 |
| | Specificity | 0.95 | 0.05 |
| | Positive Likelihood ratio | 20.00 | |
| | Negative Likelihood ratio | 0.00 | |
| | Negative predictive value | 1.00 | 0.00 |
| | Positive predictive value | 0.95 | 0.05 |
| | Accuracy | 0.975 | |
| DT | | Estimate | Standard Error |
| | Sensitivity | 0.95 | 0.05 |
| | Specificity | 0.90 | 0.07 |
| | Positive Likelihood ratio | 9.50 | |
| | Negative Likelihood ratio | 0.06 | |
| | Negative predictive value | 0.95 | 0.05 |
| | Positive predictive value | 0.90 | 0.06 |
| | Accuracy | 0.925 | |
| RF | | Estimate | Standard Error |
| | Sensitivity | 0.90 | 0.07 |
| | Specificity | 0.95 | 0.05 |
| | Positive Likelihood ratio | 18.00 | |
| | Negative Likelihood ratio | 0.11 | |
| | Negative predictive value | 0.90 | 0.06 |
| | Positive predictive value | 0.95 | 0.05 |
| | Accuracy | 0.925 | |
| GBT | | Estimate | Standard Error |
| | Sensitivity | 0.85 | 0.08 |
| | Specificity | 0.95 | 0.05 |
| | Positive Likelihood ratio | 17.00 | |
| | Negative Likelihood ratio | 0.16 | |
| | Negative predictive value | 0.86 | 0.07 |
| | Positive predictive value | 0.94 | 0.05 |
| | Accuracy | 0.900 | |
| Esemble | | Estimate | Standard Error |
| | Sensitivity | 1.00 | 0.00 |
| | Specificity | 0.95 | 0.05 |
| | Positive Likelihood ratio | 20.00 | |
| | Negative Likelihood ratio | 0.00 | |
| | Negative predictive value | 1.00 | 0.00 |
| | Positive predictive value | 0.95 | 0.05 |
| | **Accuracy** | **0.975** | |

| No Feature Selection and Optimization | | | |
|---|---|---|---|
| | | Estimate | Standard Error |
| NB | Sensitivity | 1.00 | 0.00 |
| | Specificity | 0.95 | 0.05 |
| | Positive Likelihood ratio | 20.00 | |
| | Negative Likelihood ratio | 0.00 | |
| | Negative predictive value | 1.00 | 0.00 |
| | Positive predictive value | 0.95 | 0.05 |
| | Accuracy | 0.975 | |
| GLM | | Estimate | Standard Error |
| | Sensitivity | 1.00 | 0.00 |
| | Specificity | 1.00 | 0.00 |
| | Positive Likelihood ratio | #DIV/0! | |
| | Negative Likelihood ratio | 0.00 | |
| | Negative predictive value | 1.00 | 0.00 |
| | Positive predictive value | 1.00 | 0.00 |
| | Accuracy | 1.000 | |
| LR | | Estimate | Standard Error |
| | Sensitivity | 1.00 | 0.00 |
| | Specificity | 0.90 | 0.07 |
| | Positive Likelihood ratio | 10.00 | |
| | Negative Likelihood ratio | 0.00 | |
| | Negative predictive value | 1.00 | 0.00 |
| | Positive predictive value | 0.91 | 0.06 |
| | Accuracy | 0.950 | |
| FLM | | Estimate | Standard Error |
| | Sensitivity | 0.80 | 0.09 |
| | Specificity | 0.95 | 0.05 |
| | Positive Likelihood ratio | 16.00 | |
| | Negative Likelihood ratio | 0.21 | |
| | Negative predictive value | 0.83 | 0.08 |
| | Positive predictive value | 0.94 | 0.06 |
| | Accuracy | 0.875 | |
| DL | | Estimate | Standard Error |
| | Sensitivity | 0.95 | 0.05 |
| | Specificity | 0.95 | 0.05 |
| | Positive Likelihood ratio | 19.00 | |
| | Negative Likelihood ratio | 0.05 | |
| | Negative predictive value | 0.95 | 0.05 |
| | Positive predictive value | 0.95 | 0.05 |
| | Accuracy | 0.950 | |
| DT | | Estimate | Standard Error |
| | Sensitivity | 0.85 | 0.08 |
| | Specificity | 1.00 | 0.00 |
| | Positive Likelihood ratio | #DIV/0! | |
| | Negative Likelihood ratio | 0.15 | |
| | Negative predictive value | 0.87 | 0.07 |
| | Positive predictive value | 1.00 | 0.00 |
| | Accuracy | 0.925 | |
| RF | | Estimate | Standard Error |
| | Sensitivity | 0.90 | 0.07 |
| | Specificity | 0.95 | 0.05 |
| | Positive Likelihood ratio | 18.00 | |
| | Negative Likelihood ratio | 0.11 | |
| | Negative predictive value | 0.90 | 0.06 |
| | Positive predictive value | 0.95 | 0.05 |
| | Accuracy | 0.925 | |
| GBT | | Estimate | Standard Error |
| | Sensitivity | 0.85 | 0.08 |
| | Specificity | 0.95 | 0.05 |
| | Positive Likelihood ratio | 17.00 | |
| | Negative Likelihood ratio | 0.16 | |
| | Negative predictive value | 0.86 | 0.07 |
| | Positive predictive value | 0.94 | 0.05 |
| | Accuracy | 0.900 | |
| Esemble | | Estimate | Standard Error |
| | Sensitivity | 1.00 | 0.00 |
| | Specificity | 0.95 | 0.05 |
| | Positive Likelihood ratio | 20.00 | |
| | Negative Likelihood ratio | 0.00 | |
| | Negative predictive value | 1.00 | 0.00 |
| | Positive predictive value | 0.95 | 0.04 |
| | **Accuracy** | **0.976** | |

- **Dataset PT**

| Full Optimizer | | | |
|---|---|---|---|
| | | Estimate | Standard Error |
| NB | Sensitivity | 1.00 | 0.00 |
| | Specificity | 1.00 | 0.00 |
| | Positive Likelihood ratio | #DIV/0! | |
| | Negative Likelihood ratio | 0.00 | |
| | Negative predictive value | 1.00 | 0.00 |
| | Positive predictive value | 1.00 | 0.00 |
| | Accuracy | 1.000 | |
| | | Estimate | Standard Error |
| GLM | Sensitivity | 1.00 | 0.00 |
| | Specificity | 1.00 | 0.00 |
| | Positive Likelihood ratio | #DIV/0! | |
| | Negative Likelihood ratio | 0.00 | |
| | Negative predictive value | 1.00 | 0.00 |
| | Positive predictive value | 1.00 | 0.00 |
| | Accuracy | 1.000 | |
| | | Estimate | Standard Error |
| LR | Sensitivity | 1.00 | 0.00 |
| | Specificity | 0.95 | 0.05 |
| | Positive Likelihood ratio | 20.00 | |
| | Negative Likelihood ratio | 0.00 | |
| | Negative predictive value | 1.00 | 0.00 |
| | Positive predictive value | 0.95 | 0.05 |
| | Accuracy | 0.975 | |
| | | Estimate | Standard Error |
| FLM | Sensitivity | 1.00 | 0.00 |
| | Specificity | 1.00 | 0.00 |
| | Positive Likelihood ratio | #DIV/0! | |
| | Negative Likelihood ratio | 0.00 | |
| | Negative predictive value | 1.00 | 0.00 |
| | Positive predictive value | 1.00 | 0.00 |
| | Accuracy | 1.000 | |
| | | Estimate | Standard Error |
| DL | Sensitivity | 1.00 | 0.00 |
| | Specificity | 1.00 | 0.00 |
| | Positive Likelihood ratio | #DIV/0! | |
| | Negative Likelihood ratio | 0.00 | |
| | Negative predictive value | 1.00 | 0.00 |
| | Positive predictive value | 1.00 | 0.00 |
| | Accuracy | 1.000 | |
| | | Estimate | Standard Error |
| DT | Sensitivity | 1.00 | 0.00 |
| | Specificity | 1.00 | 0.00 |
| | Positive Likelihood ratio | #DIV/0! | |
| | Negative Likelihood ratio | 0.00 | |
| | Negative predictive value | 1.00 | 0.00 |
| | Positive predictive value | 1.00 | 0.00 |
| | Accuracy | 1.000 | |
| | | Estimate | Standard Error |
| RF | Sensitivity | 1.00 | 0.00 |
| | Specificity | 1.00 | 0.00 |
| | Positive Likelihood ratio | #DIV/0! | |
| | Negative Likelihood ratio | 0.00 | |
| | Negative predictive value | 1.00 | 0.00 |
| | Positive predictive value | 1.00 | 0.00 |
| | Accuracy | 1.000 | |
| | | Estimate | Standard Error |
| GBT | Sensitivity | 0.95 | 0.05 |
| | Specificity | 1.00 | 0.00 |
| | Positive Likelihood ratio | #DIV/0! | |
| | Negative Likelihood ratio | 0.05 | |
| | Negative predictive value | 0.95 | 0.05 |
| | Positive predictive value | 1.00 | 0.00 |
| | Accuracy | 0.975 | |
| | | Estimate | Standard Error |
| SVM | Sensitivity | 1.00 | 0.00 |
| | Specificity | 1.00 | 0.00 |
| | Positive Likelihood ratio | #DIV/0! | |
| | Negative Likelihood ratio | 0.00 | |
| | Negative predictive value | 1.00 | 0.00 |
| | Positive predictive value | 1.00 | 0.00 |
| | Accuracy | 1.000 | |
| | | Estimate | Standard Error |
| Esemble | Sensitivity | 1.00 | 0.00 |
| | Specificity | 1.00 | 0.00 |
| | Positive Likelihood ratio | #DIV/0! | |
| | Negative Likelihood ratio | 0.00 | |
| | Negative predictive value | 1.00 | 0.00 |
| | Positive predictive value | 1.00 | 0.00 |
| | **Accuracy** | **1.000** | |

| No Feature Selection | | | |
|---|---|---|---|
| | | Estimate | Standard Error |
| NB | Sensitivity | 1.00 | 0.00 |
| | Specificity | 1.00 | 0.00 |
| | Positive Likelihood ratio | #DIV/0! | |
| | Negative Likelihood ratio | 0.00 | |
| | Negative predictive value | 1.00 | 0.00 |
| | Positive predictive value | 1.00 | 0.00 |
| | Accuracy | 1.000 | |
| | | Estimate | Standard Error |
| GLM | Sensitivity | 1.00 | 0.00 |
| | Specificity | 1.00 | 0.00 |
| | Positive Likelihood ratio | #DIV/0! | |
| | Negative Likelihood ratio | 0.00 | |
| | Negative predictive value | 1.00 | 0.00 |
| | Positive predictive value | 1.00 | 0.00 |
| | Accuracy | 1.000 | |
| | | Estimate | Standard Error |
| LR | Sensitivity | 1.00 | 0.00 |
| | Specificity | 1.00 | 0.00 |
| | Positive Likelihood ratio | #DIV/0! | |
| | Negative Likelihood ratio | 0.00 | |
| | Negative predictive value | 1.00 | 0.00 |
| | Positive predictive value | 1.00 | 0.00 |
| | Accuracy | 1.000 | |
| | | Estimate | Standard Error |
| FLM | Sensitivity | 1.00 | 0.00 |
| | Specificity | 1.00 | 0.00 |
| | Positive Likelihood ratio | #DIV/0! | |
| | Negative Likelihood ratio | 0.00 | |
| | Negative predictive value | 1.00 | 0.00 |
| | Positive predictive value | 1.00 | 0.00 |
| | Accuracy | 1.000 | |
| | | Estimate | Standard Error |
| DL | Sensitivity | 0.95 | 0.05 |
| | Specificity | 1.00 | 0.00 |
| | Positive Likelihood ratio | #DIV/0! | |
| | Negative Likelihood ratio | 0.05 | |
| | Negative predictive value | 0.95 | 0.05 |
| | Positive predictive value | 1.00 | 0.00 |
| | Accuracy | 0.975 | |
| | | Estimate | Standard Error |
| DT | Sensitivity | 1.00 | 0.00 |
| | Specificity | 1.00 | 0.00 |
| | Positive Likelihood ratio | #DIV/0! | |
| | Negative Likelihood ratio | 0.00 | |
| | Negative predictive value | 1.00 | 0.00 |
| | Positive predictive value | 1.00 | 0.00 |
| | Accuracy | 1.000 | |
| | | Estimate | Standard Error |
| RF | Sensitivity | 1.00 | 0.00 |
| | Specificity | 1.00 | 0.00 |
| | Positive Likelihood ratio | #DIV/0! | |
| | Negative Likelihood ratio | 0.00 | |
| | Negative predictive value | 1.00 | 0.00 |
| | Positive predictive value | 1.00 | 0.00 |
| | Accuracy | 1.000 | |
| | | Estimate | Standard Error |
| GBT | Sensitivity | 1.00 | 0.00 |
| | Specificity | 1.00 | 0.00 |
| | Positive Likelihood ratio | #DIV/0! | |
| | Negative Likelihood ratio | 0.00 | |
| | Negative predictive value | 1.00 | 0.00 |
| | Positive predictive value | 1.00 | 0.00 |
| | Accuracy | 1.000 | |
| | | Estimate | Standard Error |
| SVM | Sensitivity | 1.00 | 0.00 |
| | Specificity | 1.00 | 0.00 |
| | Positive Likelihood ratio | #DIV/0! | |
| | Negative Likelihood ratio | 0.00 | |
| | Negative predictive value | 1.00 | 0.00 |
| | Positive predictive value | 1.00 | 0.00 |
| | Accuracy | 1.000 | |
| | | Estimate | Standard Error |
| Esemble | Sensitivity | 1.00 | 0.00 |
| | Specificity | 1.00 | 0.00 |
| | Positive Likelihood ratio | #DIV/0! | |
| | Negative Likelihood ratio | 0.00 | |
| | Negative predictive value | 1.00 | 0.00 |
| | Positive predictive value | 1.00 | 0.00 |
| | **Accuracy** | **1.000** | |

| No Optimization | | | |
|---|---|---|---|
| | | Estimate | Standard Error |
| NB | Sensitivity | 1.00 | 0.00 |
| | Specificity | 1.00 | 0.00 |
| | Positive Likelihood ratio | #DIV/0! | |
| | Negative Likelihood ratio | 0.00 | |
| | Negative predictive value | 1.00 | 0.00 |
| | Positive predictive value | 1.00 | 0.00 |
| | Accuracy | 1.000 | |
| | | Estimate | Standard Error |
| GLM | Sensitivity | 1.00 | 0.00 |
| | Specificity | 1.00 | 0.00 |
| | Positive Likelihood ratio | #DIV/0! | |
| | Negative Likelihood ratio | 0.00 | |
| | Negative predictive value | 1.00 | 0.00 |
| | Positive predictive value | 1.00 | 0.00 |
| | Accuracy | 1.000 | |
| | | Estimate | Standard Error |
| FLM | Sensitivity | 1.00 | 0.00 |
| | Specificity | 1.00 | 0.00 |
| | Positive Likelihood ratio | #DIV/0! | |
| | Negative Likelihood ratio | 0.00 | |
| | Negative predictive value | 1.00 | 0.00 |
| | Positive predictive value | 1.00 | 0.00 |
| | Accuracy | 1.000 | |
| | | Estimate | Standard Error |
| DL | Sensitivity | 0.95 | 0.05 |
| | Specificity | 1.00 | 0.00 |
| | Positive Likelihood ratio | #DIV/0! | |
| | Negative Likelihood ratio | 0.05 | |
| | Negative predictive value | 0.95 | 0.05 |
| | Positive predictive value | 1.00 | 0.00 |
| | Accuracy | 0.975 | |
| | | Estimate | Standard Error |
| DT | Sensitivity | 1.00 | 0.00 |
| | Specificity | 1.00 | 0.00 |
| | Positive Likelihood ratio | #DIV/0! | |
| | Negative Likelihood ratio | 0.00 | |
| | Negative predictive value | 1.00 | 0.00 |
| | Positive predictive value | 1.00 | 0.00 |
| | Accuracy | 1.000 | |
| | | Estimate | Standard Error |
| RF | Sensitivity | 0.90 | 0.07 |
| | Specificity | 1.00 | 0.00 |
| | Positive Likelihood ratio | #DIV/0! | |
| | Negative Likelihood ratio | 0.10 | |
| | Negative predictive value | 0.91 | 0.06 |
| | Positive predictive value | 1.00 | 0.00 |
| | Accuracy | 0.950 | |
| | | Estimate | Standard Error |
| GBT | Sensitivity | 1.00 | 0.00 |
| | Specificity | 1.00 | 0.00 |
| | Positive Likelihood ratio | #DIV/0! | |
| | Negative Likelihood ratio | 0.00 | |
| | Negative predictive value | 1.00 | 0.00 |
| | Positive predictive value | 1.00 | 0.00 |
| | Accuracy | 1.000 | |
| | | Estimate | Standard Error |
| SVM | Sensitivity | 1.00 | 0.00 |
| | Specificity | 0.90 | 0.07 |
| | Positive Likelihood ratio | 10.00 | |
| | Negative Likelihood ratio | 0.00 | |
| | Negative predictive value | 1.00 | 0.00 |
| | Positive predictive value | 0.91 | 0.06 |
| | Accuracy | 0.950 | |
| | | Estimate | Standard Error |
| Esemble | Sensitivity | 1.00 | 0.00 |
| | Specificity | 1.00 | 0.00 |
| | Positive Likelihood ratio | #DIV/0! | |
| | Negative Likelihood ratio | 0.00 | |
| | Negative predictive value | 1.00 | 0.00 |
| | Positive predictive value | 1.00 | 0.00 |
| | **Accuracy** | **1.000** | |

| No Feature Selection and Optimization | | | |
|---|---|---|---|
| | | Estimate | Standard Error |
| NB | Sensitivity | 1.00 | 0.00 |
| | Specificity | 1.00 | 0.00 |
| | Positive Likelihood ratio | #DIV/0! | |
| | Negative Likelihood ratio | 0.00 | |
| | Negative predictive value | 1.00 | 0.00 |
| | Positive predictive value | 1.00 | 0.00 |
| | Accuracy | 1.000 | |
| | | Estimate | Standard Error |
| GLM | Sensitivity | 1.00 | 0.00 |
| | Specificity | 1.00 | 0.00 |
| | Positive Likelihood ratio | #DIV/0! | |
| | Negative Likelihood ratio | 0.00 | |
| | Negative predictive value | 1.00 | 0.00 |
| | Positive predictive value | 1.00 | 0.00 |
| | Accuracy | 1.000 | |
| | | Estimate | Standard Error |
| LR | Sensitivity | 1.00 | 0.00 |
| | Specificity | 1.00 | 0.00 |
| | Positive Likelihood ratio | #DIV/0! | |
| | Negative Likelihood ratio | 0.00 | |
| | Negative predictive value | 1.00 | 0.00 |
| | Positive predictive value | 1.00 | 0.00 |
| | Accuracy | 1.000 | |
| | | Estimate | Standard Error |
| FLM | Sensitivity | 1.00 | 0.00 |
| | Specificity | 1.00 | 0.00 |
| | Positive Likelihood ratio | #DIV/0! | |
| | Negative Likelihood ratio | 0.00 | |
| | Negative predictive value | 1.00 | 0.00 |
| | Positive predictive value | 1.00 | 0.00 |
| | Accuracy | 1.000 | |
| | | Estimate | Standard Error |
| DL | Sensitivity | 0.95 | 0.05 |
| | Specificity | 1.00 | 0.00 |
| | Positive Likelihood ratio | #DIV/0! | |
| | Negative Likelihood ratio | 0.05 | |
| | Negative predictive value | 0.95 | 0.05 |
| | Positive predictive value | 1.00 | 0.00 |
| | Accuracy | 0.975 | |
| | | Estimate | Standard Error |
| DT | Sensitivity | 1.00 | 0.00 |
| | Specificity | 1.00 | 0.00 |
| | Positive Likelihood ratio | #DIV/0! | |
| | Negative Likelihood ratio | 0.00 | |
| | Negative predictive value | 1.00 | 0.00 |
| | Positive predictive value | 1.00 | 0.00 |
| | Accuracy | 1.000 | |
| | | Estimate | Standard Error |
| RF | Sensitivity | 1.00 | 0.00 |
| | Specificity | 1.00 | 0.00 |
| | Positive Likelihood ratio | #DIV/0! | |
| | Negative Likelihood ratio | 0.00 | |
| | Negative predictive value | 1.00 | 0.00 |
| | Positive predictive value | 1.00 | 0.00 |
| | Accuracy | 1.000 | |
| | | Estimate | Standard Error |
| GBT | Sensitivity | 1.00 | 0.00 |
| | Specificity | 1.00 | 0.00 |
| | Positive Likelihood ratio | #DIV/0! | |
| | Negative Likelihood ratio | 0.00 | |
| | Negative predictive value | 1.00 | 0.00 |
| | Positive predictive value | 1.00 | 0.00 |
| | Accuracy | 1.000 | |
| | | Estimate | Standard Error |
| SVM | Sensitivity | 0.70 | 0.10 |
| | Specificity | 1.00 | 0.00 |
| | Positive Likelihood ratio | #DIV/0! | |
| | Negative Likelihood ratio | 0.30 | |
| | Negative predictive value | 0.77 | 0.08 |
| | Positive predictive value | 1.00 | 0.00 |
| | Accuracy | 0.850 | |
| | | Estimate | Standard Error |
| Esemble | Sensitivity | 1.00 | 0.00 |
| | Specificity | 1.00 | 0.00 |
| | Positive Likelihood ratio | #DIV/0! | |
| | Negative Likelihood ratio | 0.00 | |
| | Negative predictive value | 1.00 | 0.00 |
| | Positive predictive value | 1.00 | 0.00 |
| | **Accuracy** | **1.000** | |

- **Dataset ST-PT**

| Full Optimizer | | | |
|---|---|---|---|
| | | Estimate | Standard Error |
| NB | Sensitivity | 0.95 | 0.05 |
| | Specificity | 0.95 | 0.05 |
| | Positive Likelihood ratio | 19.00 | |
| | Negative Likelihood ratio | 0.05 | |
| | Negative predictive value | 0.95 | 0.05 |
| | Positive predictive value | 0.95 | 0.05 |
| | Accuracy | 0.950 | |
| | | Estimate | Standard Error |
| GLM | Sensitivity | 1.00 | 0.00 |
| | Specificity | 0.95 | 0.05 |
| | Positive Likelihood ratio | 20.00 | |
| | Negative Likelihood ratio | 0.00 | |
| | Negative predictive value | 1.00 | 0.00 |
| | Positive predictive value | 0.95 | 0.05 |
| | Accuracy | 0.975 | |
| | | Estimate | Standard Error |
| FLM | Sensitivity | 1.00 | 0.00 |
| | Specificity | 0.95 | 0.05 |
| | Positive Likelihood ratio | 20.00 | |
| | Negative Likelihood ratio | 0.00 | |
| | Negative predictive value | 1.00 | 0.00 |
| | Positive predictive value | 0.95 | 0.05 |
| | Accuracy | 0.975 | |
| | | Estimate | Standard Error |
| DL | Sensitivity | 1.00 | 0.00 |
| | Specificity | 1.00 | 0.00 |
| | Positive Likelihood ratio | #DIV/0! | |
| | Negative Likelihood ratio | 0.00 | |
| | Negative predictive value | 1.00 | 0.00 |
| | Positive predictive value | 1.00 | 0.00 |
| | Accuracy | 1.000 | |
| | | Estimate | Standard Error |
| DT | Sensitivity | 0.95 | 0.05 |
| | Specificity | 0.95 | 0.05 |
| | Positive Likelihood ratio | 19.00 | |
| | Negative Likelihood ratio | 0.05 | |
| | Negative predictive value | 0.95 | 0.05 |
| | Positive predictive value | 0.95 | 0.05 |
| | Accuracy | 0.950 | |
| | | Estimate | Standard Error |
| RF | Sensitivity | 1.00 | 0.00 |
| | Specificity | 0.95 | 0.05 |
| | Positive Likelihood ratio | 20.00 | |
| | Negative Likelihood ratio | 0.00 | |
| | Negative predictive value | 1.00 | 0.00 |
| | Positive predictive value | 0.95 | 0.05 |
| | Accuracy | 0.975 | |
| | | Estimate | Standard Error |
| GBT | Sensitivity | 1.00 | 0.00 |
| | Specificity | 0.95 | 0.05 |
| | Positive Likelihood ratio | 20.00 | |
| | Negative Likelihood ratio | 0.00 | |
| | Negative predictive value | 1.00 | 0.00 |
| | Positive predictive value | 0.95 | 0.05 |
| | Accuracy | 0.975 | |
| | | Estimate | Standard Error |
| Esemble | Sensitivity | 1.00 | 0.00 |
| | Specificity | 1.00 | 0.00 |
| | Positive Likelihood ratio | #DIV/0! | |
| | Negative Likelihood ratio | 0.00 | |
| | Negative predictive value | 1.00 | 0.00 |
| | Positive predictive value | 1.00 | 0.00 |
| | **Accuracy** | **1.000** | |

| No Feature Selection | | | |
|---|---|---|---|
| | | Estimate | Standard Error |
| NB | Sensitivity | 1.00 | 0.00 |
| | Specificity | 0.95 | 0.05 |
| | Positive Likelihood ratio | 20.00 | |
| | Negative Likelihood ratio | 0.00 | |
| | Negative predictive value | 1.00 | 0.00 |
| | Positive predictive value | 0.95 | 0.05 |
| | Accuracy | 0.975 | |
| | | Estimate | Standard Error |
| GLM | Sensitivity | 1.00 | 0.00 |
| | Specificity | 1.00 | 0.00 |
| | Positive Likelihood ratio | #DIV/0! | |
| | Negative Likelihood ratio | 0.00 | |
| | Negative predictive value | 1.00 | 0.00 |
| | Positive predictive value | 1.00 | 0.00 |
| | Accuracy | 1.000 | |
| | | Estimate | Standard Error |
| LR | Sensitivity | 1.00 | 0.00 |
| | Specificity | 1.00 | 0.00 |
| | Positive Likelihood ratio | #DIV/0! | |
| | Negative Likelihood ratio | 0.00 | |
| | Negative predictive value | 1.00 | 0.00 |
| | Positive predictive value | 1.00 | 0.00 |
| | Accuracy | 1.000 | |
| | | Estimate | Standard Error |
| FLM | Sensitivity | 1.00 | 0.00 |
| | Specificity | 0.95 | 0.05 |
| | Positive Likelihood ratio | 20.00 | |
| | Negative Likelihood ratio | 0.00 | |
| | Negative predictive value | 1.00 | 0.00 |
| | Positive predictive value | 0.95 | 0.05 |
| | Accuracy | 0.975 | |
| | | Estimate | Standard Error |
| DL | Sensitivity | 1.00 | 0.00 |
| | Specificity | 1.00 | 0.00 |
| | Positive Likelihood ratio | #DIV/0! | |
| | Negative Likelihood ratio | 0.00 | |
| | Negative predictive value | 1.00 | 0.00 |
| | Positive predictive value | 1.00 | 0.00 |
| | Accuracy | 1.000 | |
| | | Estimate | Standard Error |
| DT | Sensitivity | 1.00 | 0.00 |
| | Specificity | 0.95 | 0.05 |
| | Positive Likelihood ratio | 20.00 | |
| | Negative Likelihood ratio | 0.00 | |
| | Negative predictive value | 1.00 | 0.00 |
| | Positive predictive value | 0.95 | 0.05 |
| | Accuracy | 0.975 | |
| | | Estimate | Standard Error |
| RF | Sensitivity | 1.00 | 0.00 |
| | Specificity | 0.95 | 0.05 |
| | Positive Likelihood ratio | 20.00 | |
| | Negative Likelihood ratio | 0.00 | |
| | Negative predictive value | 1.00 | 0.00 |
| | Positive predictive value | 0.95 | 0.05 |
| | Accuracy | 0.975 | |
| | | Estimate | Standard Error |
| GBT | Sensitivity | 1.00 | 0.00 |
| | Specificity | 1.00 | 0.00 |
| | Positive Likelihood ratio | #DIV/0! | |
| | Negative Likelihood ratio | 0.00 | |
| | Negative predictive value | 1.00 | 0.00 |
| | Positive predictive value | 1.00 | 0.00 |
| | Accuracy | 1.000 | |
| | | Estimate | Standard Error |
| SVM | Sensitivity | 0.95 | 0.05 |
| | Specificity | 1.00 | 0.00 |
| | Positive Likelihood ratio | #DIV/0! | |
| | Negative Likelihood ratio | 0.05 | |
| | Negative predictive value | 0.95 | 0.05 |
| | Positive predictive value | 1.00 | 0.00 |
| | Accuracy | 0.975 | |
| | | Estimate | Standard Error |
| Esemble | Sensitivity | 1.00 | 0.00 |
| | Specificity | 1.00 | 0.00 |
| | Positive Likelihood ratio | #DIV/0! | |
| | Negative Likelihood ratio | 0.00 | |
| | Negative predictive value | 1.00 | 0.00 |
| | Positive predictive value | 1.00 | 0.00 |
| | **Accuracy** | **1.000** | |

| No Optimization | | | |
|---|---|---|---|
| NB | | Estimate | Standard Error |
| | Sensitivity | 1.00 | 0.00 |
| | Specificity | 0.95 | 0.05 |
| | Positive Likelihood ratio | 20.00 | |
| | Negative Likelihood ratio | 0.00 | |
| | Negative predictive value | 1.00 | 0.00 |
| | Positive predictive value | 0.95 | 0.05 |
| | Accuracy | 0.975 | |
| GLM | | Estimate | Standard Error |
| | Sensitivity | 1.00 | 0.00 |
| | Specificity | 1.00 | 0.00 |
| | Positive Likelihood ratio | #DIV/0! | |
| | Negative Likelihood ratio | 0.00 | |
| | Negative predictive value | 1.00 | 0.00 |
| | Positive predictive value | 1.00 | 0.00 |
| | Accuracy | 1.000 | |
| LR | | Estimate | Standard Error |
| | Sensitivity | 1.00 | 0.00 |
| | Specificity | 1.00 | 0.00 |
| | Positive Likelihood ratio | #DIV/0! | |
| | Negative Likelihood ratio | 0.00 | |
| | Negative predictive value | 1.00 | 0.00 |
| | Positive predictive value | 1.00 | 0.00 |
| | Accuracy | 1.000 | |
| FLM | | Estimate | Standard Error |
| | Sensitivity | 0.95 | 0.05 |
| | Specificity | 0.95 | 0.05 |
| | Positive Likelihood ratio | 19.00 | |
| | Negative Likelihood ratio | 0.05 | |
| | Negative predictive value | 0.95 | 0.05 |
| | Positive predictive value | 0.95 | 0.05 |
| | Accuracy | 0.950 | |
| DL | | Estimate | Standard Error |
| | Sensitivity | 1.00 | 0.00 |
| | Specificity | 1.00 | 0.00 |
| | Positive Likelihood ratio | #DIV/0! | |
| | Negative Likelihood ratio | 0.00 | |
| | Negative predictive value | 1.00 | 0.00 |
| | Positive predictive value | 1.00 | 0.00 |
| | Accuracy | 1.000 | |
| DT | | Estimate | Standard Error |
| | Sensitivity | 0.95 | 0.05 |
| | Specificity | 0.95 | 0.05 |
| | Positive Likelihood ratio | 19.00 | |
| | Negative Likelihood ratio | 0.05 | |
| | Negative predictive value | 0.95 | 0.05 |
| | Positive predictive value | 0.95 | 0.05 |
| | Accuracy | 0.950 | |
| RF | | Estimate | Standard Error |
| | Sensitivity | 1.00 | 0.00 |
| | Specificity | 0.95 | 0.05 |
| | Positive Likelihood ratio | 20.00 | |
| | Negative Likelihood ratio | 0.00 | |
| | Negative predictive value | 1.00 | 0.00 |
| | Positive predictive value | 0.95 | 0.05 |
| | Accuracy | 0.975 | |
| GBT | | Estimate | Standard Error |
| | Sensitivity | 0.95 | 0.05 |
| | Specificity | 1.00 | 0.00 |
| | Positive Likelihood ratio | #DIV/0! | |
| | Negative Likelihood ratio | 0.05 | |
| | Negative predictive value | 0.95 | 0.05 |
| | Positive predictive value | 1.00 | 0.00 |
| | Accuracy | 0.975 | |
| SVM | | Estimate | Standard Error |
| | Sensitivity | 0.95 | 0.05 |
| | Specificity | 1.00 | 0.00 |
| | Positive Likelihood ratio | #DIV/0! | |
| | Negative Likelihood ratio | 0.05 | |
| | Negative predictive value | 0.95 | 0.05 |
| | Positive predictive value | 1.00 | 0.00 |
| | Accuracy | 0.975 | |
| Esemble | | Estimate | Standard Error |
| | Sensitivity | 1.00 | 0.00 |
| | Specificity | 1.00 | 0.00 |
| | Positive Likelihood ratio | #DIV/0! | |
| | Negative Likelihood ratio | 0.00 | |
| | Negative predictive value | 1.00 | 0.00 |
| | Positive predictive value | 1.00 | 0.00 |
| | **Accuracy** | **1.000** | |

| No Feature Selection and Optimization | | | |
|---|---|---|---|
| NB | | Estimate | Standard Error |
| | Sensitivity | 1.00 | 0.00 |
| | Specificity | 0.95 | 0.05 |
| | Positive Likelihood ratio | 20.00 | |
| | Negative Likelihood ratio | 0.00 | |
| | Negative predictive value | 1.00 | 0.00 |
| | Positive predictive value | 0.95 | 0.05 |
| | Accuracy | 0.975 | |
| GLM | | Estimate | Standard Error |
| | Sensitivity | 1.00 | 0.00 |
| | Specificity | 1.00 | 0.00 |
| | Positive Likelihood ratio | #DIV/0! | |
| | Negative Likelihood ratio | 0.00 | |
| | Negative predictive value | 1.00 | 0.00 |
| | Positive predictive value | 1.00 | 0.00 |
| | Accuracy | 1.000 | |
| LR | | Estimate | Standard Error |
| | Sensitivity | 1.00 | 0.00 |
| | Specificity | 1.00 | 0.00 |
| | Positive Likelihood ratio | #DIV/0! | |
| | Negative Likelihood ratio | 0.00 | |
| | Negative predictive value | 1.00 | 0.00 |
| | Positive predictive value | 1.00 | 0.00 |
| | Accuracy | 1.000 | |
| FLM | | Estimate | Standard Error |
| | Sensitivity | 1.00 | 0.00 |
| | Specificity | 0.95 | 0.05 |
| | Positive Likelihood ratio | 20.00 | |
| | Negative Likelihood ratio | 0.00 | |
| | Negative predictive value | 1.00 | 0.00 |
| | Positive predictive value | 0.95 | 0.05 |
| | Accuracy | 0.975 | |
| DL | | Estimate | Standard Error |
| | Sensitivity | 1.00 | 0.00 |
| | Specificity | 0.95 | 0.05 |
| | Positive Likelihood ratio | 20.00 | |
| | Negative Likelihood ratio | 0.00 | |
| | Negative predictive value | 1.00 | 0.00 |
| | Positive predictive value | 0.95 | 0.05 |
| | Accuracy | 0.975 | |
| DT | | Estimate | Standard Error |
| | Sensitivity | 0.95 | 0.05 |
| | Specificity | 0.95 | 0.05 |
| | Positive Likelihood ratio | 19.00 | |
| | Negative Likelihood ratio | 0.05 | |
| | Negative predictive value | 0.95 | 0.05 |
| | Positive predictive value | 0.95 | 0.05 |
| | Accuracy | 0.950 | |
| RF | | Estimate | Standard Error |
| | Sensitivity | 1.00 | 0.00 |
| | Specificity | 1.00 | 0.00 |
| | Positive Likelihood ratio | #DIV/0! | |
| | Negative Likelihood ratio | 0.00 | |
| | Negative predictive value | 1.00 | 0.00 |
| | Positive predictive value | 1.00 | 0.00 |
| | Accuracy | 1.000 | |
| GBT | | Estimate | Standard Error |
| | Sensitivity | 0.95 | 0.05 |
| | Specificity | 0.95 | 0.05 |
| | Positive Likelihood ratio | 19.00 | |
| | Negative Likelihood ratio | 0.05 | |
| | Negative predictive value | 0.95 | 0.05 |
| | Positive predictive value | 0.95 | 0.05 |
| | Accuracy | 0.950 | |
| Esemble | | Estimate | Standard Error |
| | Sensitivity | 1.00 | 0.00 |
| | Specificity | 1.00 | 0.00 |
| | Positive Likelihood ratio | #DIV/0! | |
| | Negative Likelihood ratio | 0.00 | |
| | Negative predictive value | 1.00 | 0.00 |
| | Positive predictive value | 1.00 | 0.00 |
| | **Accuracy** | **1.000** | |

## - Dataset Salmonella

| Full Optimizer | | | |
|---|---|---|---|
| | | Estimate | Standard Error |
| NB | Sensitivity | 1.00 | 0.00 |
| | Specificity | 1.00 | 0.00 |
| | Positive Likelihood ratio | #DIV/0! | |
| | Negative Likelihood ratio | 0.00 | |
| | Negative predictive value | 1.00 | 0.00 |
| | Positive predictive value | 1.00 | 0.00 |
| | Accuracy | 1.000 | |
| | | Estimate | Standard Error |
| GLM | Sensitivity | 1.00 | 0.00 |
| | Specificity | 0.90 | 0.07 |
| | Positive Likelihood ratio | 10.00 | |
| | Negative Likelihood ratio | 0.00 | |
| | Negative predictive value | 1.00 | 0.00 |
| | Positive predictive value | 0.91 | 0.06 |
| | Accuracy | 0.950 | |
| | | Estimate | Standard Error |
| FLM | Sensitivity | 0.70 | 0.10 |
| | Specificity | 0.95 | 0.05 |
| | Positive Likelihood ratio | 14.00 | |
| | Negative Likelihood ratio | 0.32 | |
| | Negative predictive value | 0.76 | 0.09 |
| | Positive predictive value | 0.93 | 0.06 |
| | Accuracy | 0.825 | |
| | | Estimate | Standard Error |
| DL | Sensitivity | 1.00 | 0.00 |
| | Specificity | 1.00 | 0.00 |
| | Positive Likelihood ratio | #DIV/0! | |
| | Negative Likelihood ratio | 0.00 | |
| | Negative predictive value | 1.00 | 0.00 |
| | Positive predictive value | 1.00 | 0.00 |
| | Accuracy | 1.000 | |
| | | Estimate | Standard Error |
| DT | Sensitivity | 0.90 | 0.07 |
| | Specificity | 0.70 | 0.10 |
| | Positive Likelihood ratio | 3.00 | |
| | Negative Likelihood ratio | 0.14 | |
| | Negative predictive value | 0.88 | 0.08 |
| | Positive predictive value | 0.75 | 0.09 |
| | Accuracy | 0.800 | |
| | | Estimate | Standard Error |
| RF | Sensitivity | 0.95 | 0.05 |
| | Specificity | 0.90 | 0.07 |
| | Positive Likelihood ratio | 9.50 | |
| | Negative Likelihood ratio | 0.06 | |
| | Negative predictive value | 0.95 | 0.05 |
| | Positive predictive value | 0.90 | 0.06 |
| | Accuracy | 0.925 | |
| | | Estimate | Standard Error |
| GBT | Sensitivity | 1.00 | 0.00 |
| | Specificity | 0.95 | 0.05 |
| | Positive Likelihood ratio | 20.00 | |
| | Negative Likelihood ratio | 0.00 | |
| | Negative predictive value | 1.00 | 0.00 |
| | Positive predictive value | 0.95 | 0.05 |
| | Accuracy | 0.975 | |
| | | Estimate | Standard Error |
| SVM | Sensitivity | 0.50 | 0.11 |
| | Specificity | 0.95 | 0.05 |
| | Positive Likelihood ratio | 10.00 | |
| | Negative Likelihood ratio | 0.53 | |
| | Negative predictive value | 0.66 | 0.09 |
| | Positive predictive value | 0.91 | 0.09 |
| | Accuracy | 0.725 | |
| | | Estimate | Standard Error |
| Esemble | Sensitivity | 1.00 | 0.00 |
| | Specificity | 1.00 | 0.00 |
| | Positive Likelihood ratio | #DIV/0! | |
| | Negative Likelihood ratio | 0.00 | |
| | Negative predictive value | 1.00 | 0.00 |
| | Positive predictive value | 1.00 | 0.00 |
| | **Accuracy** | **1.000** | |

| No Feature Selection | | | |
|---|---|---|---|
| | | Estimate | Standard Error |
| NB | Sensitivity | 1.00 | 0.00 |
| | Specificity | 0.90 | 0.07 |
| | Positive Likelihood ratio | 10.00 | |
| | Negative Likelihood ratio | 0.00 | |
| | Negative predictive value | 1.00 | 0.00 |
| | Positive predictive value | 0.91 | 0.06 |
| | Accuracy | 0.950 | |
| | | Estimate | Standard Error |
| GLM | Sensitivity | 1.00 | 0.00 |
| | Specificity | 0.90 | 0.07 |
| | Positive Likelihood ratio | 10.00 | |
| | Negative Likelihood ratio | 0.00 | |
| | Negative predictive value | 1.00 | 0.00 |
| | Positive predictive value | 0.91 | 0.06 |
| | Accuracy | 0.950 | |
| | | Estimate | Standard Error |
| LR | Sensitivity | 1.00 | 0.00 |
| | Specificity | 0.90 | 0.07 |
| | Positive Likelihood ratio | 10.00 | |
| | Negative Likelihood ratio | 0.00 | |
| | Negative predictive value | 1.00 | 0.00 |
| | Positive predictive value | 0.91 | 0.06 |
| | Accuracy | 0.950 | |
| | | Estimate | Standard Error |
| FLM | Sensitivity | 1.00 | 0.00 |
| | Specificity | 0.95 | 0.05 |
| | Positive Likelihood ratio | 20.00 | |
| | Negative Likelihood ratio | 0.00 | |
| | Negative predictive value | 1.00 | 0.00 |
| | Positive predictive value | 0.95 | 0.05 |
| | Accuracy | 0.975 | |
| | | Estimate | Standard Error |
| DL | Sensitivity | 1.00 | 0.00 |
| | Specificity | 0.95 | 0.05 |
| | Positive Likelihood ratio | 20.00 | |
| | Negative Likelihood ratio | 0.00 | |
| | Negative predictive value | 1.00 | 0.00 |
| | Positive predictive value | 0.95 | 0.05 |
| | Accuracy | 0.975 | |
| | | Estimate | Standard Error |
| DT | Sensitivity | 0.95 | 0.05 |
| | Specificity | 0.95 | 0.05 |
| | Positive Likelihood ratio | 19.00 | |
| | Negative Likelihood ratio | 0.05 | |
| | Negative predictive value | 0.95 | 0.05 |
| | Positive predictive value | 0.95 | 0.05 |
| | Accuracy | 0.950 | |
| | | Estimate | Standard Error |
| RF | Sensitivity | 1.00 | 0.00 |
| | Specificity | 0.85 | 0.08 |
| | Positive Likelihood ratio | 6.67 | |
| | Negative Likelihood ratio | 0.00 | |
| | Negative predictive value | 1.00 | 0.00 |
| | Positive predictive value | 0.87 | 0.07 |
| | Accuracy | 0.925 | |
| | | Estimate | Standard Error |
| GBT | Sensitivity | 0.95 | 0.05 |
| | Specificity | 0.95 | 0.05 |
| | Positive Likelihood ratio | 19.00 | |
| | Negative Likelihood ratio | 0.05 | |
| | Negative predictive value | 0.95 | 0.05 |
| | Positive predictive value | 0.95 | 0.05 |
| | Accuracy | 0.950 | |
| | | Estimate | Standard Error |
| SVM | Sensitivity | 1.00 | 0.00 |
| | Specificity | 0.95 | 0.05 |
| | Positive Likelihood ratio | 20.00 | |
| | Negative Likelihood ratio | 0.00 | |
| | Negative predictive value | 1.00 | 0.00 |
| | Positive predictive value | 0.95 | 0.05 |
| | Accuracy | 0.975 | |
| | | Estimate | Standard Error |
| Esemble | Sensitivity | 0.95 | 0.05 |
| | Specificity | 1.00 | 0.00 |
| | Positive Likelihood ratio | #DIV/0! | |
| | Negative Likelihood ratio | 0.05 | |
| | Negative predictive value | 0.95 | 0.05 |
| | Positive predictive value | 1.00 | 0.00 |
| | **Accuracy** | **0.975** | |

| No Optimization | | | |
|---|---|---|---|
| | | Estimate | Standard Error |
| NB | Sensitivity | 1.00 | 0.00 |
| | Specificity | 1.00 | 0.00 |
| | Positive Likelihood ratio | #DIV/0! | |
| | Negative Likelihood ratio | 0.00 | |
| | Negative predictive value | 1.00 | 0.00 |
| | Positive predictive value | 1.00 | 0.00 |
| | Accuracy | 1.000 | |
| | | Estimate | Standard Error |
| GLM | Sensitivity | 1.00 | 0.00 |
| | Specificity | 1.00 | 0.00 |
| | Positive Likelihood ratio | #DIV/0! | |
| | Negative Likelihood ratio | 0.00 | |
| | Negative predictive value | 1.00 | 0.00 |
| | Positive predictive value | 1.00 | 0.00 |
| | Accuracy | 1.000 | |
| | | Estimate | Standard Error |
| LR | Sensitivity | 1.00 | 0.00 |
| | Specificity | 1.00 | 0.00 |
| | Positive Likelihood ratio | #DIV/0! | |
| | Negative Likelihood ratio | 0.00 | |
| | Negative predictive value | 1.00 | 0.00 |
| | Positive predictive value | 1.00 | 0.00 |
| | Accuracy | 1.000 | |
| | | Estimate | Standard Error |
| FLM | Sensitivity | 0.80 | 0.09 |
| | Specificity | 0.85 | 0.08 |
| | Positive Likelihood ratio | 5.33 | |
| | Negative Likelihood ratio | 0.24 | |
| | Negative predictive value | 0.81 | 0.09 |
| | Positive predictive value | 0.84 | 0.08 |
| | Accuracy | 0.825 | |
| | | Estimate | Standard Error |
| DL | Sensitivity | 1.00 | 0.00 |
| | Specificity | 1.00 | 0.00 |
| | Positive Likelihood ratio | #DIV/0! | |
| | Negative Likelihood ratio | 0.00 | |
| | Negative predictive value | 1.00 | 0.00 |
| | Positive predictive value | 1.00 | 0.00 |
| | Accuracy | 1.000 | |
| | | Estimate | Standard Error |
| DT | Sensitivity | 0.90 | 0.07 |
| | Specificity | 0.75 | 0.10 |
| | Positive Likelihood ratio | 3.60 | |
| | Negative Likelihood ratio | 0.13 | |
| | Negative predictive value | 0.88 | 0.08 |
| | Positive predictive value | 0.78 | 0.09 |
| | Accuracy | 0.825 | |
| | | Estimate | Standard Error |
| RF | Sensitivity | 0.90 | 0.07 |
| | Specificity | 0.95 | 0.05 |
| | Positive Likelihood ratio | 18.00 | |
| | Negative Likelihood ratio | 0.11 | |
| | Negative predictive value | 0.90 | 0.06 |
| | Positive predictive value | 0.95 | 0.05 |
| | Accuracy | 0.925 | |
| | | Estimate | Standard Error |
| GBT | Sensitivity | 0.95 | 0.05 |
| | Specificity | 0.95 | 0.05 |
| | Positive Likelihood ratio | 19.00 | |
| | Negative Likelihood ratio | 0.05 | |
| | Negative predictive value | 0.95 | 0.05 |
| | Positive predictive value | 0.95 | 0.05 |
| | Accuracy | 0.950 | |
| | | Estimate | Standard Error |
| SVM | Sensitivity | 0.55 | 0.11 |
| | Specificity | 0.95 | 0.05 |
| | Positive Likelihood ratio | 11.00 | |
| | Negative Likelihood ratio | 0.47 | |
| | Negative predictive value | 0.68 | 0.09 |
| | Positive predictive value | 0.92 | 0.08 |
| | Accuracy | 0.750 | |
| | | Estimate | Standard Error |
| Esemble | Sensitivity | 1.00 | 0.00 |
| | Specificity | 1.00 | 0.00 |
| | Positive Likelihood ratio | #DIV/0! | |
| | Negative Likelihood ratio | 0.00 | |
| | Negative predictive value | 1.00 | 0.00 |
| | Positive predictive value | 1.00 | 0.00 |
| | **Accuracy** | **1.000** | |

| No Feature Selection and Optimization | | | |
|---|---|---|---|
| | | Estimate | Standard Error |
| NB | Sensitivity | 1.00 | 0.00 |
| | Specificity | 0.90 | 0.07 |
| | Positive Likelihood ratio | 10.00 | |
| | Negative Likelihood ratio | 0.00 | |
| | Negative predictive value | 1.00 | 0.00 |
| | Positive predictive value | 0.91 | 0.06 |
| | Accuracy | 0.950 | |
| | | Estimate | Standard Error |
| GLM | Sensitivity | 1.00 | 0.00 |
| | Specificity | 0.90 | 0.07 |
| | Positive Likelihood ratio | 10.00 | |
| | Negative Likelihood ratio | 0.00 | |
| | Negative predictive value | 1.00 | 0.00 |
| | Positive predictive value | 0.91 | 0.06 |
| | Accuracy | 0.950 | |
| | | Estimate | Standard Error |
| LR | Sensitivity | 1.00 | 0.00 |
| | Specificity | 0.90 | 0.07 |
| | Positive Likelihood ratio | 10.00 | |
| | Negative Likelihood ratio | 0.00 | |
| | Negative predictive value | 1.00 | 0.00 |
| | Positive predictive value | 0.91 | 0.06 |
| | Accuracy | 0.950 | |
| | | Estimate | Standard Error |
| FLM | Sensitivity | 0.85 | 0.08 |
| | Specificity | 0.95 | 0.05 |
| | Positive Likelihood ratio | 17.00 | |
| | Negative Likelihood ratio | 0.16 | |
| | Negative predictive value | 0.86 | 0.07 |
| | Positive predictive value | 0.94 | 0.05 |
| | Accuracy | 0.900 | |
| | | Estimate | Standard Error |
| DL | Sensitivity | 1.00 | 0.00 |
| | Specificity | 0.95 | 0.05 |
| | Positive Likelihood ratio | 20.00 | |
| | Negative Likelihood ratio | 0.00 | |
| | Negative predictive value | 1.00 | 0.00 |
| | Positive predictive value | 0.95 | 0.05 |
| | Accuracy | 0.975 | |
| | | Estimate | Standard Error |
| DT | Sensitivity | 1.00 | 0.00 |
| | Specificity | 0.80 | 0.09 |
| | Positive Likelihood ratio | 5.00 | |
| | Negative Likelihood ratio | 0.00 | |
| | Negative predictive value | 1.00 | 0.00 |
| | Positive predictive value | 0.83 | 0.08 |
| | Accuracy | 0.900 | |
| | | Estimate | Standard Error |
| RF | Sensitivity | 1.00 | 0.00 |
| | Specificity | 0.85 | 0.08 |
| | Positive Likelihood ratio | 6.67 | |
| | Negative Likelihood ratio | 0.00 | |
| | Negative predictive value | 1.00 | 0.00 |
| | Positive predictive value | 0.87 | 0.07 |
| | Accuracy | 0.925 | |
| | | Estimate | Standard Error |
| GBT | Sensitivity | 0.95 | 0.05 |
| | Specificity | 0.95 | 0.05 |
| | Positive Likelihood ratio | 19.00 | |
| | Negative Likelihood ratio | 0.05 | |
| | Negative predictive value | 0.95 | 0.05 |
| | Positive predictive value | 0.95 | 0.05 |
| | Accuracy | 0.950 | |
| | | Estimate | Standard Error |
| Esemble | Sensitivity | 1.00 | 0.00 |
| | Specificity | 0.95 | 0.05 |
| | Positive Likelihood ratio | 20.00 | |
| | Negative Likelihood ratio | 0.00 | |
| | Negative predictive value | 1.00 | 0.00 |
| | Positive predictive value | 0.95 | 0.05 |
| | **Accuracy** | **0.975** | |

## Pan Wang et all.

- **Dataset OC**

| Full Optimizer | | | |
|---|---|---|---|
| | | Estimate | Standard Error |
| NB | Sensitivity | 1.00 | 0.00 |
| | Specificity | 0.85 | 0.08 |
| | Positive Likelihood ratio | 6.67 | |
| | Negative Likelihood ratio | 0.00 | |
| | Negative predictive value | 1.00 | 0.00 |
| | Positive predictive value | 0.87 | 0.07 |
| | Accuracy | 0.925 | |
| | | Estimate | Standard Error |
| GLM | Sensitivity | 0.90 | 0.07 |
| | Specificity | 0.95 | 0.05 |
| | Positive Likelihood ratio | 18.00 | |
| | Negative Likelihood ratio | 0.11 | |
| | Negative predictive value | 0.90 | 0.06 |
| | Positive predictive value | 0.95 | 0.05 |
| | Accuracy | 0.925 | |
| | | Estimate | Standard Error |
| FLM | Sensitivity | 0.90 | 0.07 |
| | Specificity | 0.90 | 0.07 |
| | Positive Likelihood ratio | 9.00 | |
| | Negative Likelihood ratio | 0.11 | |
| | Negative predictive value | 0.90 | 0.07 |
| | Positive predictive value | 0.90 | 0.07 |
| | Accuracy | 0.900 | |
| | | Estimate | Standard Error |
| DL | Sensitivity | 0.80 | 0.09 |
| | Specificity | 0.95 | 0.05 |
| | Positive Likelihood ratio | 16.00 | |
| | Negative Likelihood ratio | 0.21 | |
| | Negative predictive value | 0.83 | 0.08 |
| | Positive predictive value | 0.94 | 0.06 |
| | Accuracy | 0.875 | |
| | | Estimate | Standard Error |
| DT | Sensitivity | 0.65 | 0.11 |
| | Specificity | 0.80 | 0.09 |
| | Positive Likelihood ratio | 3.25 | |
| | Negative Likelihood ratio | 0.44 | |
| | Negative predictive value | 0.70 | 0.10 |
| | Positive predictive value | 0.76 | 0.10 |
| | Accuracy | 0.725 | |
| | | Estimate | Standard Error |
| RF | Sensitivity | 0.95 | 0.05 |
| | Specificity | 0.90 | 0.07 |
| | Positive Likelihood ratio | 9.50 | |
| | Negative Likelihood ratio | 0.06 | |
| | Negative predictive value | 0.95 | 0.05 |
| | Positive predictive value | 0.90 | 0.06 |
| | Accuracy | 0.925 | |
| | | Estimate | Standard Error |
| GBT | Sensitivity | 0.90 | 0.07 |
| | Specificity | 0.90 | 0.07 |
| | Positive Likelihood ratio | 9.00 | |
| | Negative Likelihood ratio | 0.11 | |
| | Negative predictive value | 0.90 | 0.07 |
| | Positive predictive value | 0.90 | 0.07 |
| | Accuracy | 0.900 | |
| | | Estimate | Standard Error |
| SVM | Sensitivity | 1.00 | 0.00 |
| | Specificity | 0.90 | 0.07 |
| | Positive Likelihood ratio | 10.00 | |
| | Negative Likelihood ratio | 0.00 | |
| | Negative predictive value | 1.00 | 0.00 |
| | Positive predictive value | 0.91 | 0.06 |
| | Accuracy | 0.950 | |
| | | Estimate | Standard Error |
| Esemble | Sensitivity | 1.00 | 0.00 |
| | Specificity | 0.90 | 0.07 |
| | Positive Likelihood ratio | 10.00 | |
| | Negative Likelihood ratio | 0.00 | |
| | Negative predictive value | 1.00 | 0.00 |
| | Positive predictive value | 0.91 | 0.06 |
| | **Accuracy** | **0.950** | |

| No Feature Selection | | | |
|---|---|---|---|
| | | Estimate | Standard Error |
| NB | Sensitivity | 0.45 | 0.11 |
| | Specificity | 1.00 | 0.00 |
| | Positive Likelihood ratio | #DIV/0! | |
| | Negative Likelihood ratio | 0.55 | |
| | Negative predictive value | 0.65 | 0.09 |
| | Positive predictive value | 1.00 | 0.00 |
| | Accuracy | 0.725 | |
| | | Estimate | Standard Error |
| GLM | Sensitivity | 0.95 | 0.05 |
| | Specificity | 0.90 | 0.07 |
| | Positive Likelihood ratio | 9.50 | |
| | Negative Likelihood ratio | 0.06 | |
| | Negative predictive value | 0.95 | 0.05 |
| | Positive predictive value | 0.90 | 0.06 |
| | Accuracy | 0.925 | |
| | | Estimate | Standard Error |
| LR | Sensitivity | 0.85 | 0.08 |
| | Specificity | 0.90 | 0.07 |
| | Positive Likelihood ratio | 8.50 | |
| | Negative Likelihood ratio | 0.17 | |
| | Negative predictive value | 0.86 | 0.08 |
| | Positive predictive value | 0.89 | 0.07 |
| | Accuracy | 0.875 | |
| | | Estimate | Standard Error |
| FLM | Sensitivity | 0.85 | 0.08 |
| | Specificity | 0.90 | 0.07 |
| | Positive Likelihood ratio | 8.50 | |
| | Negative Likelihood ratio | 0.17 | |
| | Negative predictive value | 0.86 | 0.08 |
| | Positive predictive value | 0.89 | 0.07 |
| | Accuracy | 0.875 | |
| | | Estimate | Standard Error |
| DL | Sensitivity | 0.95 | 0.05 |
| | Specificity | 0.95 | 0.05 |
| | Positive Likelihood ratio | 19.00 | |
| | Negative Likelihood ratio | 0.05 | |
| | Negative predictive value | 0.95 | 0.05 |
| | Positive predictive value | 0.95 | 0.05 |
| | Accuracy | 0.950 | |
| | | Estimate | Standard Error |
| DT | Sensitivity | 0.60 | 0.11 |
| | Specificity | 1.00 | 0.00 |
| | Positive Likelihood ratio | #DIV/0! | |
| | Negative Likelihood ratio | 0.40 | |
| | Negative predictive value | 0.71 | 0.09 |
| | Positive predictive value | 1.00 | 0.00 |
| | Accuracy | 0.800 | |
| | | Estimate | Standard Error |
| RF | Sensitivity | 0.95 | 0.05 |
| | Specificity | 0.85 | 0.08 |
| | Positive Likelihood ratio | 6.33 | |
| | Negative Likelihood ratio | 0.06 | |
| | Negative predictive value | 0.94 | 0.05 |
| | Positive predictive value | 0.86 | 0.07 |
| | Accuracy | 0.900 | |
| | | Estimate | Standard Error |
| GBT | Sensitivity | 0.70 | 0.10 |
| | Specificity | 0.95 | 0.05 |
| | Positive Likelihood ratio | 14.00 | |
| | Negative Likelihood ratio | 0.32 | |
| | Negative predictive value | 0.76 | 0.09 |
| | Positive predictive value | 0.93 | 0.06 |
| | Accuracy | 0.825 | |
| | | Estimate | Standard Error |
| SVM | Sensitivity | 0.95 | 0.05 |
| | Specificity | 0.90 | 0.07 |
| | Positive Likelihood ratio | 9.50 | |
| | Negative Likelihood ratio | 0.06 | |
| | Negative predictive value | 0.95 | 0.05 |
| | Positive predictive value | 0.90 | 0.06 |
| | Accuracy | 0.925 | |
| | | Estimate | Standard Error |
| Esemble | Sensitivity | 1.00 | 0.00 |
| | Specificity | 0.90 | 0.07 |
| | Positive Likelihood ratio | 10.00 | |
| | Negative Likelihood ratio | 0.00 | |
| | Negative predictive value | 1.00 | 0.00 |
| | Positive predictive value | 0.91 | 0.06 |
| | **Accuracy** | **0.950** | |

| No Optimization | | | |
|---|---|---|---|
| NB | | Estimate | Standard Error |
| | Sensitivity | 0.45 | 0.11 |
| | Specificity | 1.00 | 0.00 |
| | Positive Likelihood ratio | #DIV/0! | |
| | Negative Likelihood ratio | 0.55 | |
| | Negative predictive value | 0.65 | 0.09 |
| | Positive predictive value | 1.00 | 0.00 |
| | Accuracy | 0.725 | |
| GLM | | Estimate | Standard Error |
| | Sensitivity | 0.90 | 0.07 |
| | Specificity | 0.95 | 0.05 |
| | Positive Likelihood ratio | 18.00 | |
| | Negative Likelihood ratio | 0.11 | |
| | Negative predictive value | 0.90 | 0.06 |
| | Positive predictive value | 0.95 | 0.05 |
| | Accuracy | 0.925 | |
| FLM | | Estimate | Standard Error |
| | Sensitivity | 0.85 | 0.08 |
| | Specificity | 0.85 | 0.08 |
| | Positive Likelihood ratio | 5.67 | |
| | Negative Likelihood ratio | 0.18 | |
| | Negative predictive value | 0.85 | 0.08 |
| | Positive predictive value | 0.85 | 0.08 |
| | Accuracy | 0.850 | |
| DL | | Estimate | Standard Error |
| | Sensitivity | 0.90 | 0.07 |
| | Specificity | 0.90 | 0.07 |
| | Positive Likelihood ratio | 9.00 | |
| | Negative Likelihood ratio | 0.11 | |
| | Negative predictive value | 0.90 | 0.07 |
| | Positive predictive value | 0.90 | 0.07 |
| | Accuracy | 0.900 | |
| DT | | Estimate | Standard Error |
| | Sensitivity | 0.80 | 0.09 |
| | Specificity | 0.90 | 0.07 |
| | Positive Likelihood ratio | 8.00 | |
| | Negative Likelihood ratio | 0.22 | |
| | Negative predictive value | 0.82 | 0.08 |
| | Positive predictive value | 0.89 | 0.07 |
| | Accuracy | 0.850 | |
| RF | | Estimate | Standard Error |
| | Sensitivity | 0.75 | 0.10 |
| | Specificity | 0.95 | 0.05 |
| | Positive Likelihood ratio | 15.00 | |
| | Negative Likelihood ratio | 0.26 | |
| | Negative predictive value | 0.79 | 0.08 |
| | Positive predictive value | 0.94 | 0.06 |
| | Accuracy | 0.850 | |
| GBT | | Estimate | Standard Error |
| | Sensitivity | 0.85 | 0.08 |
| | Specificity | 0.50 | 0.11 |
| | Positive Likelihood ratio | 1.70 | |
| | Negative Likelihood ratio | 0.30 | |
| | Negative predictive value | 0.77 | 0.12 |
| | Positive predictive value | 0.63 | 0.09 |
| | Accuracy | 0.675 | |
| SVM | | Estimate | Standard Error |
| | Sensitivity | 0.90 | 0.07 |
| | Specificity | 0.90 | 0.07 |
| | Positive Likelihood ratio | 9.00 | |
| | Negative Likelihood ratio | 0.11 | |
| | Negative predictive value | 0.90 | 0.07 |
| | Positive predictive value | 0.90 | 0.07 |
| | Accuracy | 0.900 | |
| Esemble | | Estimate | Standard Error |
| | Sensitivity | 1.00 | 0.00 |
| | Specificity | 0.85 | 0.08 |
| | Positive Likelihood ratio | 6.67 | |
| | Negative Likelihood ratio | 0.00 | |
| | Negative predictive value | 1.00 | 0.00 |
| | Positive predictive value | 0.87 | 0.07 |
| | **Accuracy** | **0.925** | |

| No Feature Selection and Optimization | | | |
|---|---|---|---|
| NB | | Estimate | Standard Error |
| | Sensitivity | 0.45 | 0.11 |
| | Specificity | 1.00 | 0.00 |
| | Positive Likelihood ratio | #DIV/0! | |
| | Negative Likelihood ratio | 0.55 | |
| | Negative predictive value | 0.65 | 0.09 |
| | Positive predictive value | 1.00 | 0.00 |
| | Accuracy | 0.725 | |
| GLM | | Estimate | Standard Error |
| | Sensitivity | 0.95 | 0.05 |
| | Specificity | 0.90 | 0.07 |
| | Positive Likelihood ratio | 9.50 | |
| | Negative Likelihood ratio | 0.06 | |
| | Negative predictive value | 0.95 | 0.05 |
| | Positive predictive value | 0.90 | 0.06 |
| | Accuracy | 0.925 | |
| LR | | Estimate | Standard Error |
| | Sensitivity | 0.85 | 0.08 |
| | Specificity | 0.90 | 0.07 |
| | Positive Likelihood ratio | 8.50 | |
| | Negative Likelihood ratio | 0.17 | |
| | Negative predictive value | 0.86 | 0.08 |
| | Positive predictive value | 0.89 | 0.07 |
| | Accuracy | 0.875 | |
| FLM | | Estimate | Standard Error |
| | Sensitivity | 0.85 | 0.08 |
| | Specificity | 0.90 | 0.07 |
| | Positive Likelihood ratio | 8.50 | |
| | Negative Likelihood ratio | 0.17 | |
| | Negative predictive value | 0.86 | 0.08 |
| | Positive predictive value | 0.89 | 0.07 |
| | Accuracy | 0.875 | |
| DL | | Estimate | Standard Error |
| | Sensitivity | 0.95 | 0.05 |
| | Specificity | 0.95 | 0.05 |
| | Positive Likelihood ratio | 19.00 | |
| | Negative Likelihood ratio | 0.05 | |
| | Negative predictive value | 0.95 | 0.05 |
| | Positive predictive value | 0.95 | 0.05 |
| | Accuracy | 0.950 | |
| DT | | Estimate | Standard Error |
| | Sensitivity | 0.70 | 0.10 |
| | Specificity | 0.95 | 0.05 |
| | Positive Likelihood ratio | 14.00 | |
| | Negative Likelihood ratio | 0.32 | |
| | Negative predictive value | 0.76 | 0.09 |
| | Positive predictive value | 0.93 | 0.06 |
| | Accuracy | 0.825 | |
| RF | | Estimate | Standard Error |
| | Sensitivity | 1.00 | 0.00 |
| | Specificity | 0.90 | 0.07 |
| | Positive Likelihood ratio | 10.00 | |
| | Negative Likelihood ratio | 0.00 | |
| | Negative predictive value | 1.00 | 0.00 |
| | Positive predictive value | 0.91 | 0.06 |
| | Accuracy | 0.950 | |
| GBT | | Estimate | Standard Error |
| | Sensitivity | 0.80 | 0.09 |
| | Specificity | 0.65 | 0.11 |
| | Positive Likelihood ratio | 2.29 | |
| | Negative Likelihood ratio | 0.31 | |
| | Negative predictive value | 0.76 | 0.10 |
| | Positive predictive value | 0.70 | 0.10 |
| | Accuracy | 0.725 | |
| SVM | | Estimate | Standard Error |
| | Sensitivity | 1.00 | 0.00 |
| | Specificity | 0.90 | 0.07 |
| | Positive Likelihood ratio | 10.00 | |
| | Negative Likelihood ratio | 0.00 | |
| | Negative predictive value | 1.00 | 0.00 |
| | Positive predictive value | 0.91 | 0.06 |
| | Accuracy | 0.950 | |
| Esemble | | Estimate | Standard Error |
| | Sensitivity | 1.00 | 0.00 |
| | Specificity | 0.90 | 0.07 |
| | Positive Likelihood ratio | 10.00 | |
| | Negative Likelihood ratio | 0.00 | |
| | Negative predictive value | 1.00 | 0.00 |
| | Positive predictive value | 0.91 | 0.06 |
| | **Accuracy** | **0.950** | |
