# Supplementary material for: Double-Weighted Bayesian Model Combination for Metabolomics Data Description and Prediction
Source: Metabolites. 2025 Mar 21;15(4):214. doi: 10.3390/metabo15040214 (PMC12029032; doi:10.3390/metabo15040214)
Supplement: Supplementary file 1 [file metabolites-15-00214-s001.zip › metabolites-3488782-supplementary/Supplementary S3.pdf]

Supplementary 3: Ablation Study on Synthetic Data Augmentation and Class Imbalance Approaches"

Dataset considered from Arturas Grauslys et all.: CV with SMOTE vs CV without SMOTE

With SMOTE			
NB		Estimate	Standard Error
	Sensitivity	0.80	0.09
	Specificity	0.68	0.10
	Positive Likelihood ratio	2.51	
	Negative Likelihood ratio	0.29	
	Negative predictive value	0.79	0.09
	Positive predictive value	0.70	0.10
	Accuracy	0.738	
GLM		Estimate	Standard Error
	Sensitivity	0.75	0.10
	Specificity	0.91	0.06
	Positive Likelihood ratio	8.25	
	Negative Likelihood ratio	0.28	
	Negative predictive value	0.80	0.08
	Positive predictive value	0.88	0.08
	Accuracy	0.833	
FLM		Estimate	Standard Error
	Sensitivity	0.75	0.10
	Specificity	0.95	0.04
	Positive Likelihood ratio	16.50	
	Negative Likelihood ratio	0.26	
	Negative predictive value	0.81	0.08
	Positive predictive value	0.94	0.06
	Accuracy	0.857	
DL		Estimate	Standard Error
	Sensitivity	0.60	0.11
	Specificity	0.95	0.04
	Positive Likelihood ratio	13.20	
	Negative Likelihood ratio	0.42	
	Negative predictive value	0.72	0.08
	Positive predictive value	0.92	0.07
	Accuracy	0.786	
DT		Estimate	Standard Error
	Sensitivity	0.45	0.11
	Specificity	0.95	0.04
	Positive Likelihood ratio	9.90	
	Negative Likelihood ratio	0.58	
	Negative predictive value	0.66	0.08
	Positive predictive value	0.90	0.09
	Accuracy	0.714	
RF		Estimate	Standard Error
	Sensitivity	0.35	0.11
	Specificity	0.91	0.06
	Positive Likelihood ratio	3.85	
	Negative Likelihood ratio	0.72	
	Negative predictive value	0.61	0.09
	Positive predictive value	0.78	0.14
	Accuracy	0.643	
GBT		Estimate	Standard Error
	Sensitivity	0.50	0.11
	Specificity	0.73	0.09
	Positive Likelihood ratio	1.83	
	Negative Likelihood ratio	0.69	
	Negative predictive value	0.62	0.10
	Positive predictive value	0.63	0.12
	Accuracy	0.619	
SVM		Estimate	Standard Error
	Sensitivity	0.70	0.10
	Specificity	0.77	0.09
	Positive Likelihood ratio	3.08	
	Negative Likelihood ratio	0.39	
	Negative predictive value	0.74	0.09
	Positive predictive value	0.74	0.10
	Accuracy	0.738	
Esemble		Estimate	Standard Error
	Sensitivity	0.85	0.08
	Specificity	0.91	0.06
	Positive Likelihood ratio	9.35	
	Negative Likelihood ratio	0.17	
	Negative predictive value	0.87	0.07
	Positive predictive value	0.89	0.07
	**Accuracy**	**0.881**	

Without SMOTE			
NB		Estimate	Standard Error
	Sensitivity	0.86	0.13
	Specificity	0.76	0.07
	Positive Likelihood ratio	3.54	
	Negative Likelihood ratio	0.19	
	Negative predictive value	0.96	0.04
	Positive predictive value	0.43	0.13
	Accuracy	0.775	
GLM		Estimate	Standard Error
	Sensitivity	0.59	0.10
	Specificity	0.94	0.05
	Positive Likelihood ratio	10.64	
	Negative Likelihood ratio	0.43	
	Negative predictive value	0.65	0.09
	Positive predictive value	0.93	0.07
	Accuracy	0.750	
DL		Estimate	Standard Error
	Sensitivity	0.67	0.11
	Specificity	0.91	0.06
	Positive Likelihood ratio	7.33	
	Negative Likelihood ratio	0.37	
	Negative predictive value	0.77	0.08
	Positive predictive value	0.86	0.09
	Accuracy	0.800	
DT		Estimate	Standard Error
	Sensitivity	0.70	0.14
	Specificity	0.77	0.08
	Positive Likelihood ratio	3.00	
	Negative Likelihood ratio	0.39	
	Negative predictive value	0.88	0.06
	Positive predictive value	0.50	0.13
	Accuracy	0.750	
RF		Estimate	Standard Error
	Sensitivity	0.87	0.09
	Specificity	0.96	0.04
	Positive Likelihood ratio	21.67	
	Negative Likelihood ratio	0.14	
	Negative predictive value	0.92	0.05
	Positive predictive value	0.93	0.07
	Accuracy	0.925	
GBT		Estimate	Standard Error
	Sensitivity	0.46	0.09
	Specificity	0.92	0.08
	Positive Likelihood ratio	5.57	
	Negative Likelihood ratio	0.58	
	Negative predictive value	0.42	0.10
	Positive predictive value	0.93	0.07
	Accuracy	0.600	
Esemble		Estimate	Standard Error
	Sensitivity	0.86	0.09
	Specificity	0.88	0.06
	Positive Likelihood ratio	7.43	
	Negative Likelihood ratio	0.16	
	Negative predictive value	0.92	0.05
	Positive predictive value	0.80	0.10
	**Accuracy**	**0.875**	
